# Nanoparticles Based on Cross-Linked Poly(Lipoic Acid) Protect Macrophages and Cardiomyocytes from Oxidative Stress and Ischemia Reperfusion Injury

**DOI:** 10.3390/antiox11050907

**Published:** 2022-05-05

**Authors:** Chiara Bellini, Salvatore Antonucci, Lucía Morillas-Becerril, Sara Scarpa, Regina Tavano, Fabrizio Mancin, Fabio Di Lisa, Emanuele Papini

**Affiliations:** 1 Department of Biomedical Sciences, University of Padova, Via U. Bassi 58/b, 35121 Padova, Italy; chiara.bellini@ttk.elte.hu (C.B.); salvatore.antonucci@unipd.it (S.A.); sara.scarpa@studenti.unipd.it (S.S.); regina.tavano@unipd.it (R.T.); 2 CRIBI—Centre for Innovative Biotechnology Research, University of Padova, Via U. Bassi 58/b, 35121 Padova, Italy; 3 Department of Chemical Sciences, University of Padova, Via F. Marzolo 1, 35121 Padova, Italy; lucia.morillasbecerril@unipd.it (L.M.-B.); fabrizio.mancin@unipd.it (F.M.)

**Keywords:** poly(lipoic acid), nanoparticles, ischemia and reperfusion injury

## Abstract

The control of radical damage and oxidative stress, phenomena involved in a large number of human pathologies, is a major pharmaceutical and medical goal. We here show that two biocompatible formulations of Pluronic-stabilized, poly (lipoic acid)-based nanoparticles (NP) effectively antagonized the formation of radicals and reactive oxygen species (ROS). These NPs, not only intrinsically scavenged radicals in a-cellular DPPH/ABTS assays, but also inhibited the overproduction of ROS induced by tert-Butyl hydroperoxide (t-BHP) in tumor cells (HeLa), human macrophages and neonatal rat ventricular myocytes (NRVMs). NPs were captured by macrophages and cardiomyocytes much more effectively as compared to HeLa cells and non-phagocytic leukocytes, eventually undergoing intracellular disassembly. Notably, NPs decreased the mitochondrial ROS generation induced by simulated Ischemia/Reperfusion Injury (IRI) in isolated cardiomyocytes. NPs also prevented IRI-triggered cardiomyocyte necrosis, mitochondrial dysfunction, and alterations of contraction-related intracellular Ca^2+^ waves. Hence, NPs appear to be an effective and cardiomyocyte-selective drug to protect against damages induced by post-ischemic reperfusion.

## 1. Introduction

Tissue and cell damage induced by high levels of radicals and reactive oxygen species (ROS) is linked to several pathological conditions and intoxications [1,2,3,4,5]. In this context, so-called Ischemia/Reperfusion Injury (IRI) has great clinical relevance. This is severe damage which can affect major organs, like the liver, heart, lungs, intestine, brain or kidneys. In particular, the damage is exacerbated by the restoration of blood flow (i.e., reperfusion) after a prolonged absence of tissue perfusion (i.e., ischemia), as reported in myocardial infarction, ischemic stroke, or in liver transplantations) [6]. Among the many mechanisms involved in IRI, a large increase in mitochondrial ROS formation is considered a hallmark and a major event responsible for causing tissue damage and death [7,8]. Indeed, although there are multiple potential sources of ROS in IRI, mitochondria represent a major one. They are abundant in the heart, and they contain several sites involved in ROS production (e.g., respiratory chain Complex I via Reverse Electron Transport or RET; monoamine oxidases, the translocation of p66Shc protein and the Permeability Transition Pore) [9].

Several agonists have been proposed to protect from oxidative stress and IRI [10,11]. Some of them have direct ROS scavenging actions, while others act by inducing an Nrf2-dependent increase in antioxidant defenses, resulting in anti-inflammatory and cytoprotective effects. However, despite positive results in experimental studies, such as in the case of cyclosporine A (CyA), no relevant candidates have emerged from clinical trials. This failure is likely due to several reasons. Some candidates target inflammation or the permeability transition pore (PTP), a protein complex of still uncertain molecular identity, involved in several diseases, including cardiac ischemia, and targeted by cyclophilin D and other inhibitors [12,13]. However, these agents tackle events downstream of reperfusion-induced oxidative stress leaving ROS formation unaltered [14,15,16,17]. This limitation is likely to be overridden by administration of small molecules with ROS scavenging ability. However, small drugs in general suffer from rapid clearance and low target specificity.

The nanoparticle (NP) approach may present special advantages compared to small drugs, for example, due to the possibility of reducing renal clearance, prolonging drug circulation, selectively targeting specific cells, and delivering the therapeutic principle only to the desired tissue and cells [18,19,20,21,22,23]. Most NP-based agents proposed for protecting against IRI are drug-delivery systems, where NPs act as biocompatible carriers of pharmacologically active compounds. For example, PLGA-NPs, albumin-based, or chitosan NPs were loaded with PTP inhibitors [24,25] or with antioxidant agents, such as quercetin, resveratrol or gallic acid [26,27,28,29,30,31]. However, in the case of PLGA-nanoparticles, it was demonstrated that the uptake by cardiomyocytes occurs after the insurgence of the oxidative stress deriving from the ischemic injury, and it was likely due to the cell damage induced by the oxidative stress. In several other cases, the intrinsic radical-scavenging action of the material forming the NPs, such as cerium dioxide, selenium, gold and carbon nanotubes, was exploited to counteract IRI oxidative stress [32,33]. However, the toxicological effects and the biocompatibility of these materials are likely to undermine the achievement of positive results [34,35]. These examples demonstrate the need for more efficient, selective, and biocompatible nanosystems for effective treatment of IRI.

Poly(lipoic acid) polymers and nanoparticles are an emerging class of biodegradable vectors capable to respond to local tissues environment [36,37]. The inclusion of disulfide units from lipoic acid in the nanoparticles’ structure, translates in their redox-responsive behavior and eventual cargo release in presence of reducing agents, as observed for other particles [38,39,40]. We recently proposed a new concept of polylipoic acid-based NPs obtained by nanoprecipitation of di- or tri-lipoic acid derivatives in the presence of a pluronic surfactant followed by a Ring Opening Disulphide polymerization (RODEP) [41]. The resulting nanoparticles feature average diameters in the 100–200 nm range and a PEGylated surface, formed by the pluronic surfactant, which remains entangled in the polymeric matrix of the core. PEG coating is a well-recognized way to improve the colloidal stability of NPs and their biocompatibility and pharmacokinetics properties compared to equivalent uncoated NPs. Indeed, these NPs were found to be very stable in water and saline solutions. In addition, in vitro biological studies showed that these nanoparticles displayed both a reduced interaction with plasma proteins, including opsonins, and high biocompatibility. We also demonstrated that polylipoic nanoparticles underwent degradation, by thiol-induced depolymerization [42], in the presence of endogenous reducing agents such as glutathione, which is present in relatively high concentrations in the cells’ cytosol, but not in the extracellular fluids. Results from in vivo experiments confirmed the low toxicity and revealed the ability of these nanoparticles to rapidly accumulate in the rat heart after injection. These observations suggested the possibility to use these poly-lipoic nanoparticles to contrast oxidative stress, and in particular IRI, in different cell lines due to their potential antioxidant activity. Indeed, not only are the lipoic acid molecules released upon nanoparticle degradation/reduction known antioxidants, but the disulphide bonds ubiquitous in the nanoparticle polymeric matrix before degradation can also have a ROS scavenging activity.

On these premises, we decided to investigate the potential of poly(lipoic acid) nanoparticles for scavenging free radicals in an abiotic measurement system to determine maximum antioxidant capacity while also testing in cellular types representative of the milieu encountered in organ-level ischemia. In particular, we used macrophages and NRVMs as a recognized and reliable model, keeping several crucial functional and structural features of adult cardiomyocytes from oxidative stress and IRI.

## 2. Materials and Methods

### 2.1. Cells

HeLa cells (obtained from DSB University of Padova, Padova, Italy) were maintained in Dulbecco’s Modified Eagle Medium (DMEM, Gibco, Thermo Fisher Scientific, Waltham, MA, USA) supplemented with 10% FCS (*v/v*) (Euroclone^®^, Pero (MI), Italy) and antibiotics (penicillin and streptomycin, Invitrogen) at 37 °C in a humidified atmosphere containing 5% (*v/v*) CO_2_; cells were split every 2–3 days. 

Human macrophages were obtained from human monocytes, purified from buffy coats of healthy donors as previously described [43] by two sequential centrifugations on Ficoll and Percoll (GE Healthcare, Chicago, IL, USA) gradients, respectively, and differentiated for 7 days with 100 ng/mL macrophage colony-stimulating factor (M-CSF, BD Biosciences, Franklin Lakes, NJ, USA ) in RPMI-1640 medium supplemented with 20% FCS (*v/v*) at 37 °C in a humidified atmosphere containing 5% (*v/v*) CO_2_. The M2 phenotype was routinely assessed by *CD14, CD16, Cd80 and Cd163* expression. The peripheral blood mononuclear cells utilized in this study were derived from buffy coats obtained from healthy blood donors and anonymously provided by the Transfusion Centre of the Hospital of Padova. Written informed consent for the use of the buffy coats for research purposes was obtained from blood donors by the Transfusion Centre. Data related to human samples were all analyzed anonymously. Human leukocytes, provided by the Transfusion Centre of the Hospital of Padova, were obtained not as a consequence of experimentation on human beings, but as a consequence of voluntary and informed blood donation for transfusions; no approval of an ethics committee is needed in such cases at our institution. 

Neonatal rat ventricular myocytes (NRVMs) were isolated from 1 to 3 days old Wistar rats as described previously [44]. Cardiomyocytes were plated in 0.1% porcine gelatin (Sigma-Aldrich, St Louis, MO, USA) coated plates at variable density (at least 2.5 × 10^5^ cells/mL) in MEM supplemented with 10% FBS (*v/v*) (Thermo Fisher Scientific, Waltham, MA, USA), 1% penicillin/streptomycin (*v/v*) (Thermo Fisher Scientific), 1% non-essential amino acids (*v/v*) (Thermo Fisher Scientific, Waltham, MA, USA), 1 mM 5-Bromo-2-Deoxyuridine (Sigma-Aldrich, St Louis, MO, USA). Cells were maintained at 37 °C in the presence of 5% CO_2_. The medium was changed to MEM supplemented with 1% FBS (*v/v*), 1% penicillin/streptomycin (*v/v*), and 1% non-essential amino acids (*v/v*) after 24 h of plating. The procedures relative to the isolation of primary cardiomyocytes from neonatal rats were approved by the local ethical committee of the University of Padova (Organism for Animal Welfare, OPBA) and by the Italian Ministry of Health (Ethical Approval Number: D2784.N.7RX).

### 2.2. DPPH Assay and ABTS Assay

To assess the intrinsic scavenging capacity of NPs DPPH and ABTS assays were performed. First, 100 μL of NPs at different concentrations (up to 400 μg/mL) were added to methanol buffer solution (60% methanol, 40% 0.1 M acetate buffer *v/v*, pH 5.5) were added to 50 µM DPPH radical (1,1-diphenyl-2-picrylhydrazyl) solution, and changes in optical density at 517 nm were measured every 120 s for 60 times. Results are indicated as percentage of antioxidant capacity inhibition (% DPPH scavenging). 7 mM ABTS (2,2’-azino-bis(3-ethylbenzothiazoline-6-sulfonic acid) solution and 2.46 mM potassium persulfate were mixed together and maintained for 18 h in the dark to obtain the radical molecule ABTS•+. Then, 100 μL of NPs at different concentrations (up to 400 μg/mL) in PBS solution were added to 0.28 mM ABTS•+ solution, and changes in optical density at 734 nm were measured every 60 s for 40 times at 30 °C. Results are indicated as percentage of antioxidant capacity inhibition (% ABTS scavenging). Butylated hydroxytoluene and water were used as positive and negative controls, respectively, in both assays.

### 2.3. Cellular Uptake Analysis

Cells were seeded into 24-well plates (5 × 10^4^ cells/well HeLa, 2 × 10^6^ cells/well human macrophages, 1.5 × 10^5^ cells/well NRVM). On the day of the experiment, cells were treated with different concentrations of fluorescent-doped NPs (up to 400 μg/mL) in medium supplemented with 10% of FCS or HS; cells incubated with no nanoparticles were used as a negative control. Then, cells were washed in PBS, detached, centrifuged (1500 rpm, 5 min) and resuspended in FACS buffer (PBS containing 1% FCS and 0.1% NaN_3_). Samples were analyzed by flow cytometry (BD FACSCantoII) and data processed through BD FACSDiva™ Software (BD). 

### 2.4. Pulse–Chase Assay

For the pulse experiment, 2 × 10^6^ macrophages were incubated with 400 µg/mL of F127@**1**-NP and F127@**2**-NP for different incubation times (5′, 10′, 20′, 40′, 60′, 120′ and 180′); cells were then washed with PBS, harvested, resuspended in FACS buffer (PBS supplemented with 1% FBS) and analyzed by flow cytometry. For the chase experiment, macrophages were incubated with 400 µg/mL of F127@**1**-NP and F127@**2**-NP for 10′; cells were then washed twice with PBS and incubated for different times with 10% FCS RPMI without nanoparticles; cells were then recovered, washed again with PBS, resuspended in FACS buffer and analyzed by flow cytometry.

NRVMs were seeded in either 24-well (pulse) or 96-well (chase) plates at density of 3 × 10^5^ cells/mL. A pulse of 100 µM fluorescent-doped NPs was applied to cells at different timings, ranging from 5 min to 180 min. At the end of the pulse protocol, cells were harvested and resuspended in FACS buffer. Samples were analyzed by flow cytometry and data processed through BD FACSDiva™ Software (BD). Untreated cell fluorescence was subtracted from each sample and values are expressed as Mean Fluorescence Intensity (MFI).

For two given pulse periods (i.e., 5 min and 20 min), the chase phase was evaluated in NRVMs at different time points, ranging from 10 min to 180 min. Fluorescence was monitored using a plate reader (Tecan Infinite^®^ M200 PRO). Data have been expressed as MFI normalized to basal value. 

### 2.5. Estimation of ROS Production

ROS generation in cell systems was estimated using the fluorogenic probe CM-H_2_DCFDA (Molecular Probes, Thermo Fisher Scientific, Waltham, MA, USA). Cells (1 × 10^4^ cells/well HeLa, 1 × 10^5^ cells/well human macrophages, 3 × 10^4^ cells/well NRVM) were seeded in a 96-well plate and treated with NPs at different concentrations (up to 200 μg/mL) at different time points. Then, cells were washed in PBS and loaded with a 25 µM dye for 1 h in the dark at 37 °C. Afterwards, the cells were washed and incubated with tert-Butyl hydroperoxide (t-BHP, Sigma-Aldrich, St Louis, MO, USA) at different concentrations (up to 200 µM). The fluorescence increase was estimated on a plate reader (Tecan Infinite^®^ M200 PRO) at 485 nm (λ excitation) and 527 nm (λ emission) for 1.5 h (2.5 h in NRVM experiments) [45].

### 2.6. Live Imaging

Experiments were carried out in HBSS at pH 7.4 (adjusted with NaOH) and at 37 °C. Images were acquired using an inverted fluorescence microscope (Leica DMI6000B equipped with DFC365FX camera) with PL APO 40x/1.25 oil objective. Fluorescence intensity was quantified using the Fiji distribution of the Java-based image processing program ImageJ [46], and background signal was subtracted from all analyzed regions of interest. For Ca^2+^ imaging, traces were analyzed using the “Peak Analyzer” tool of Origin Pro 9.1. The experiments were performed in the presence or absence of 100 μg/mL NPs.

Ischemic injury was simulated in vitro as previously described [44]. Briefly, a 15 mm circular glass coverslip was placed on the center of cardiomyocyte monolayer to reduce availability of nutrients and O_2_, thus inducing ischemic conditions. Reperfusion was simulated by removing the coverslip after 1 h of simulated ischemia.

To monitor mitochondrial ROS formation in NRVMs, cells were transfected with the genetically encoded H_2_O_2_ sensor Mito-HyPer (Evrogen, Moscow, Russia) [47]. NRVMs were plated on l-Plate 2-well black plates (Ibidi, Gräfelfing, Bayer, Germany ) at a density of 1 × 10^5^ cells/mL and transfected with Lipofectamine 3000 reagent (Sigma-Aldrich, St Louis, MO, USA). For each transfection, 2 μg of plasmid was diluted in 125 μL of Opti-MEM medium (Thermo Fisher Scientific) in the presence of 4 μL of P3000 reagent (Thermo Fisher Scientific, Waltham, MA, USA), and later combined with 5 μL of Lipofectamine 3000 (Thermo Fisher Scientific, Waltham, MA, USA). The DNA–lipid complexes were added to the cells and incubated at 37 °C in the presence of 5% CO_2_. Transfected cells were used for experiments after 48 h. Images were collected before the ischemic injury and in the first 5′ after the reperfusion injury. Fluorescence values were expressed as HyPer ratio (488/410 nm). 

To monitor mitochondrial membrane potential (∆Ψm), cells were incubated with 25 nM tetramethyl rhodamine (TMRM, Thermo Fisher Scientific, Waltham, MA, USA) and 1.6 μM cyclosporin H for 30 min at 37 °C, in the presence of 5% CO_2_. Cyclosporin H is necessary to block multidrug resistance glycoprotein (MDR) that otherwise would cause the release of TMRM into the extracellular space [48]. TMRM fluorescence intensity was monitored, and images were acquired during the whole experiment. Fluorescence values were normalized to basal value.

To monitor intracellular [Ca^2+^] homeostasis, cells were incubated with 5 μM Fluo-4 AM ester (Thermo Fisher Scientific, Waltham, MA, USA), 0.01% *w/v* pluronic F-127 (Sigma-Aldrich, St Louis, MO, USA), and 250 μM sulfinpyrazone (Sigma-Aldrich, St Louis, MO, USA), for 20 min at 37 °C in a humidified incubator, followed by 20 min of de-esterification. Images have been collected before the ischemic injury and in the first minute after the reperfusion injury. Since the accumulation of Fluo-4 in NRVMs can vary from different preparations, data were normalized to the DMSO control. 

### 2.7. Assessment of Cell Death

For anoxia/reoxygenation experiments, NRVMs were seeded in 24-well plates at density of 3 × 10^5^ cells/mL and incubated in 118 mM NaCl, 5 mM KCl, 1.2 mM KH_2_PO_4_, 1.2 mM MgSO_4_, 2 mM CaCl_2_, 25 mM MOPS at pH 6.4 during anoxia or pH 7.4 during reoxygenation [44]. Cells were pre-treated with different concentrations of NPs (ranging from 10 to 200 μg/mL) and 2 μM CsA or 10 μM MitoTempo were used as positive controls. Anoxia was induced by adding 10 mM 2-deoxy-D-glucose (2-DG) and incubating cells in a BD GasPak™ EZ Anaerobe Gas-generating Pouch System with an indicator (BD Biosciences, Franklin Lakes, NJ, USA) at 37 °C for 12 h [44]. To induce reoxygenation, plates were removed from the GasPak™ pouch, 2-DG was replaced with 10 mM D-glucose, the pH was restored at 7.4. The plates were then incubated for 4 h in a humidified incubator at 37 °C. The release of LDH from NRVMs was measured to evaluate cell death in anoxia, reoxygenation and during the pre-treatment phase [49]. Supernatant aliquots were collected at every time point (3 h of pre-treatment, 12 h of anoxia, and 4 h of reoxygenation), and at the end of each experiment, intact cells were lysed by incubating them with 1% Triton X-100 (Sigma-Aldrich, St Louis, MO, USA) for 30 min. Between the collection of the reoxygenation supernatant and the lysis, cells were washed with PBS 1X to remove reoxygenation-derived LDH that could interfere with the analysis of the lysate.

### 2.8. Data Analysis

All values are expressed as mean ± SE. All data were normally distributed and comparison between groups was performed by either one-way or two-way ANOVA, followed by Tukey’s post hoc multiple comparison. A value of *p* < 0.05 was considered significant.

## 3. Results

### 3.1. Synthesis and Characterization of 1- and 2-Poly(Lipoic Acid) NPs

**Figure 1:**
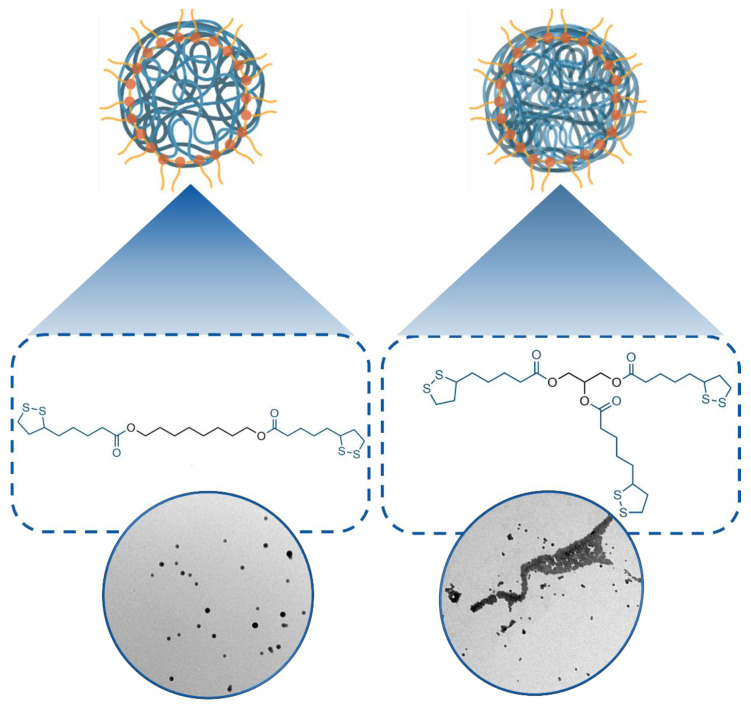
Chemical structure of the lipoic acid derivatives **1** and **2** studied in this work and their respective pluronic-stabilized poly(lipoic acid) nanoparticles.

Based on our previous screening [41], we selected derivatives **1** and **2** (Figure 1) as precursors to prepare the poly(lipoic acid) nanoparticles used in this study. They featured 1,8-octanediol and glycerol as spacers connecting respectively two and three lipoic acid molecules via ester linkages. Using these precursors, it was possible to explore the biological properties of nanoparticles characterized by different degrees of crosslinking. In particular, previous investigations revealed that less crosslinked F127@**1**-NP are more susceptible to degradation by endogenous thiols than F127@**2**-NP. The Rhodamine B/lipoic acid conjugate **3** was used, when necessary, as co-precursor for the fluorescent labeling of the nanoparticles [41]. Compounds **1**, **2** and **3** were prepared in good yields from commercially available precursors by standard synthetic protocols, as described in detail in [41]. 

The synthesis of the nanoparticles was divided into two steps. In the first one, an acetone solution of the selected lipoic acid derivatives was injected in a water solution of the F127 Pluronic surfactant buffered at pH 7 with PBS. The diffusion of acetone in water caused the formation of nanoparticles made by the aggregated precursors, stabilized by the F127 surfactant. In the second step, the polymerization and cross-linking of precursors in the nanoparticle core were induced by addition of 1-octanethiol and quenched after 30 min by addition of iodoacetamide. The nanoparticles were eventually purified by centrifugation and resuspension in water and PBS. Dynamic light scattering (DLS) analysis revealed that the batches of F127@**1**-NP and F127@**2**-NP obtained had average hydrodynamic diameters of 170–190 nm and 140–160 nm, respectively, with size dispersions in the 10–30% range (PDI values between 0.011 and 0.13, see Table S1). Larger size of F127@**2**-NP with respect to F127@**1**-NP agrees with previous reports and was attributed to the greater hydrophobicity of **2** with respect to **1**. Accordingly, co-polymerization with fluorescent derivative **3** resulted in a marginal decrease of the average particles size. TEM analysis confirmed the formation of spherical nanoparticles. Characterization data of a representative sample are reported in Figure S1A,B.

The stability of these nanoparticles upon prolonged storage (22 days) in water was studied using DLS, TEM and fluorescence spectroscopy. Hydrodynamic size, polydispersity index (PDI) and structure of both F127@**1**-NP and F127@**2**-NP, measured by DLS and TEM, did not significantly change over the same period of time (Figure S1E,F). The possible release of the co-polymerized **3** dye was investigated by measuring the fluorescence emission of the sample after centrifugation and resuspension in fresh solvent. No relevant emission decrease was observed for rhodamine-labeled F127@**2**-NPs, also, in this case, while only a small decrease was observed in the case of rhodamine-labeled F127@**1**-NPs after 3 weeks (Figure S1C,D, respectively).

### 3.2. Biocompatibility of Poly(Lipoic Acid) NPs

Consistent with previous data [17], F127@**1**-NP and F127@**2**-NP showed no cytotoxic or membrane destabilizing effects (Figure S2A–C). To ensure the biocompatibility of the NPs, the activation of the contact-triggered coagulation in human plasma (HP) and of the complement cascade in human serum (HS) was also considered (Figure S3A,B). Noticeably, NPs did not stimulate coagulation events, while only a weak induction of the complement cascade was observed in the presence of high concentrations (i.e., 400 µg/mL) of F127@**2**-NP. Consistently, both NPs formulations were virtually devoid of a protein hard corona in fetal calf serum (FCS), HP and HS (Figure S3C).

### 3.3. Radical Scavenging Activity of Poly(Lipoic Acid) NPs Compared with Free Lipoic Acid and Pluronic Acid in A-Cellular Assays

**Figure 2:**
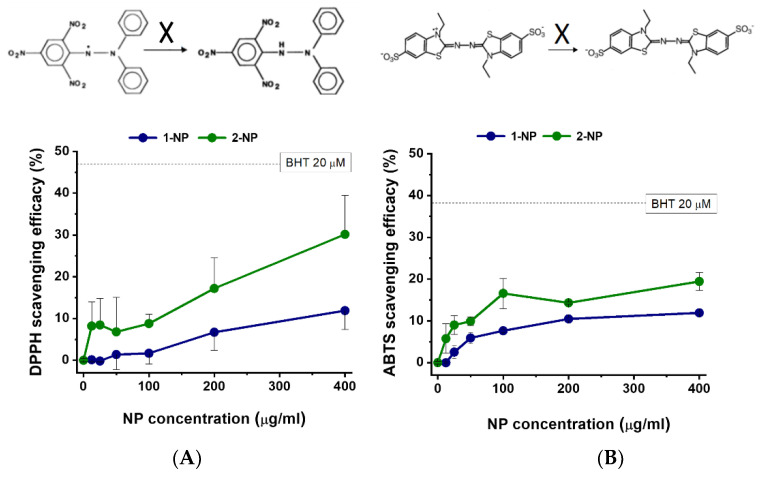
Organic radical scavenging efficacies of F127@**1**-NP and F127@**2**-NP. The indicated concentrations of NPs were incubated with DPPH (**A**) and ABTS (**B**) and the % neutralization (see top schemes) was estimated spectroscopically after 30 min incubation at 37 °C. Data are means ± SE (N = 4). The scavenging effect of BHT is indicated in the graphs for comparison.

The radical scavenging efficacy of NPs was then tested using both the DPPH and ABTS radicals. Maximal antioxidant activities of F127@**1**-NP and F127@**2**-NP at 400 µg/mL concentration against the DPPH radical in methanol rich medium were 12% and 30%, respectively (Figure 2A). This activity was lower than that of the commonly used antioxidant BHT (20 µM; 48% scavenge), but significant. Dose-response and kinetics showed that the F127@**2**-NP was about 2–3 times more effective than F127@**1**-NP as DPPH scavenger. ABTS radical scavenging experiments made it possible to assess the nanoparticles’ radical scavenging ability in a more physiological medium (PBS, pH 7.4), where the maximal activities were 12% and 19% for F127@**1**-NP and F127@**2**-NP, respectively, at 400 µg/mL concentration (Figure 2B). Again, ABTS radical neutralization by both NPs was inferior to that of the BHT positive control (38%). Dose-response and kinetics experiments showed that F127@**2**-NP was 1.4–1.7 times more effective as ABTS scavenger than F127@**1**-NP. It is interesting to note that the scavenging activity of NPs measured with the DPPH assay was better than that of their free constituents (lipoic acid and Pluronic surfactant). Conversely, in the ABTS assay, in more physiological conditions, the antiradical action of the sum of free lipoic acid and Pluronic was more effective than that of the equivalent amount of NPs (about 2 folds) (Figure S4). Conveniently, the release of Pluronic and lipoic acid is one of the results of the eventual degradation of NPs within cells after capture, suggesting that this could produce an improvement of intracellular protection from radical damage. 

### 3.4. Capture of Poly(Lipoic Acid) NPs by Different Cell Lines

Having established the radical scavenging ability of poly(lipoic acid) NPs, we investigated their internalization in different cell lines. Fluorescent labeled F127@**1**-NPs, incubated at different concentrations for 24 h, were captured by human primary macrophages and blood monocytes, and, although with reduced efficacy, by polymorphonuclear granulocytes (PMNGs). Conversely, lymphocytes captured NPs at very low levels (Figure S6A). This trend was confirmed by a time-course analysis at early times, which showed a rapid temperature-dependent initial phase of NP cell uptake, especially in macrophages (Figure S6B). 

It is worth mentioning that these results showed that the mouse macrophagic cell line RAW 264.7, being extremely ineffective at NP capture, was an unreliable model for human macrophages. 

NP association to macrophages, monocytes and lymphocytes was marginally modified when cells were incubated with FCS, HS or citrated HS (Figure S7), suggesting that these media have poor opsonic effect, in agreement with the lack of corona formation and complement activation by NPs (Figure S3B,C).

### 3.5. Capture and Antioxidative Effect of Poly(Lipoic Acid) NPs in Human Macrophage and HeLa Cells

**Figure 3:**
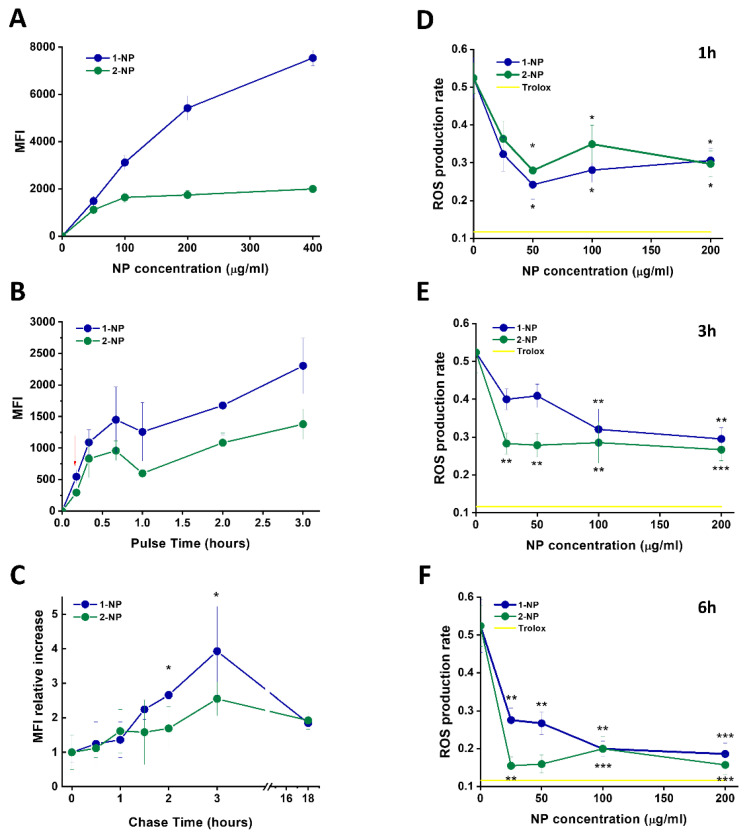
Capture, intracellular disassembly and antioxidative efficacies of F127@**1**-NP and F127@**2**-NP in t-BHP-treated human macrophages. (**A**) Cells were incubated with the indicated doses of fluorescently labeled F127@**1**-NP and F127@**2**-NP, for 3 h in DMEM plus 10% FCS and analyzed, after washing in NP-free medium, by flow cytofluorimetry. Data (Mean Fluorescence Intensity) are the mean of 4 independent experiments ± SE. (**B**) Cells were incubated with F127@**1**-NP and F127@**2**-NP (100 µg/mL) for the indicated time lapses and analyzed for their MFI by flow cytofluorimetry as above (data are mean ± SE, N = 4). The red arrow indicates the pulse–chase experiments described in panel (**C**). Cells were pulsed for 10 min as in panel (**B**), washed 3 times with PBS and further cashed in cell medium with no NPs for the indicated times. MFI acquired by flow cytometry after cell recovery are expressed as relative values compared to the MFI one after the pulse time (taken as 1). Data are mean ± SE (N = 3). * Significance t < 0.05 of the difference between F127@**2**-NP and F127@**1**-NP at a given time. (**D**–**F**) Cells were treated with the shown concentrations of F127@**1**-NP and F127@**2**-NP for 1 h (**D**), 3 h (**E**), and 6 h (**F**). After washing, cells were incubated with the ROS-sensitive probe. The ROS production was induced using the oxidative agent t-BHP (100 µM). ROS production rate (ROS production/time) is reported as a function of NP dose. Data are mean ± SE (N = 4). Statistical significance of the difference compared with no NP samples: * t < 0.05; ** t < 001; *** t < 0.001. The inhibitory effect of the positive control Trolox (500 µM) is indicated as a yellow line.

Having established that human macrophages are a good target of our NPs, the interaction of these cells with fluorescent F127@**1**-NP and F127@**2**-NP was characterized in detail. Dose-response incubation with F127@**1**-NP for 3 h (Figure 3A) resulted in a cell-associated fluorescence signal about 3–4 times more intense than that observed after incubation with F127@**2**-NP, especially at higher doses (>100 µg/mL). This behavior was only slightly modified by the presence of HS, compared to FCS, in line with weak HS interaction on these NPs (Figure S8). The results of time-course experiments indicated that the F127@**1**-NP and F127@**2**-NP capture by macrophages was quite similar at early times (<20 min). However, a divergent increase of the F127@**1**-NP signal compared to the F127@**2**-NP was detected at longer times, accounting for the observed differences after 3 h incubation (Figure 3B). In addition, time-dependent uptake profiles were clearly biphasic. To discriminate whether this late fluorescence increase both in time- and concentration-dependent experiments was due to a true differential NP capture, macrophages fluorescence was measured after a 10 min pulse with the two NPs types, followed by different chase times. Interestingly, data indicated an increase of the cell fluorescence occurring after NP endocytosis (Figure 3C) for both the nanoparticles. This behavior can only be attributed to the decrease of the self-quenching of the Rhodamine dyes, due to their release in the cell environment following NP degradation. This effect seems more evident for the F127@**1**-NP compared with F127@**2**-NP, which is in agreement with previous data showing that thiol-induced NP degradation is faster in F127@**1**-NP than in F127@**2**-NP (Figure S1E,F; see also [41]). 

Having shown that F127@**1**-NP and F127@**2**-NP are taken up by macrophages with similar efficiency, but differently degraded, we tested their antioxidant efficacy in these cells against an oxidative stress induced by the peroxide molecule t-BHP (Figure 3D–F). Pre-incubation of macrophages with NPs for 1 and 3 h reduced ROS formation upon subsequent t-BHP treatment, with an efficacy which was about half of that induced by the positive control Trolox. Interestingly, NP concentration doses as low as 25 µg/mL already gave quasi-maximal protective effects. Pre-preincubation of 6 h resulted in ROS prevention similar to that induced by Trolox (70% ROS inhibition). While the F127@**2**-NP and F127@**1**-NP anti-ROS activities in macrophages were comparable after 1-h preincubation, at longer preincubation times, F127@**2**-NP were significantly more effective.

Notably, at variance from cell-free assays, the antioxidant activity of F127@**1**-NP against t-BHP-derived reactive oxygen species (ROS) in macrophages was superior (~30% protection) than that elicited by free lipoic acid (~15%) (Figure S5). 

Analogous experiments were also performed in HeLa neoplastic cell line, where ROS production induced by t-BHP was much reduced as compared to macrophages. Although cell-associated fluorescence values were reduced about 10-fold with respect to macrophages, their relative trend was quite similar to that seen in macrophages and was similarly not influenced by HS compared to FCS. Consequently, also in these cells, NPs inhibited t-BHP-induced ROS production, with a maximal efficacy of about 60% after a 6-h preincubation (Figure S11).

### 3.6. Uptake and Antioxidative Effects of Poly(Lipoic Acid) NPs in Neonatal Rat Ventricular Myocytes (NRVMs)

Having established the antioxidant efficacy of poly(lipoic acid) NPs in phagocytes and epithelial cells, we moved to test these particles in primary murine cardiomyocytes. Indeed, the oxidative injury of cardiac cells is involved in important pathological conditions, such as IRI. 

**Figure 4:**
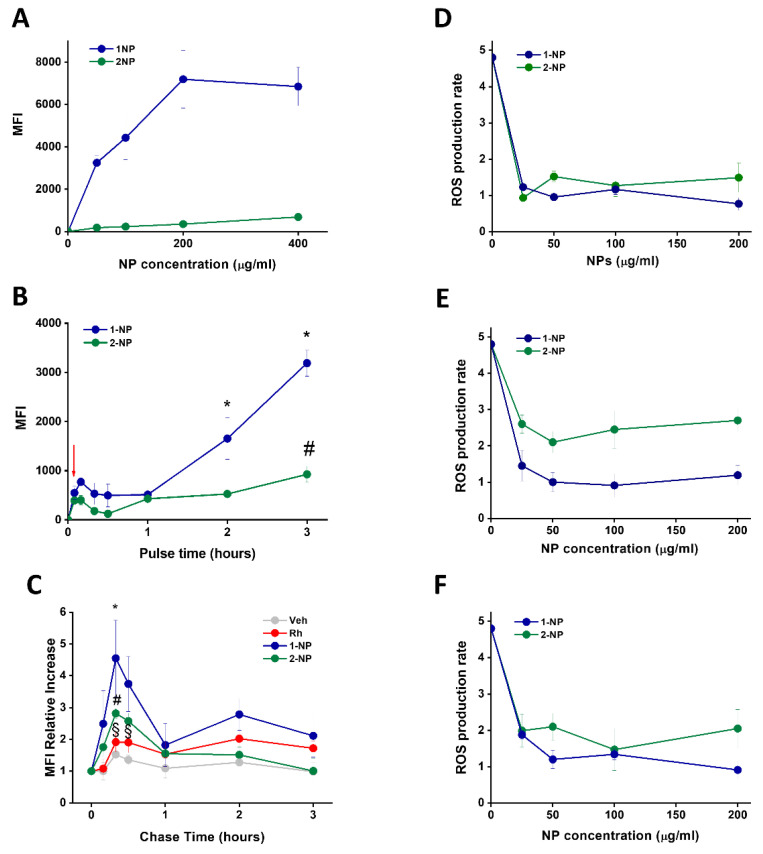
Capture, intracellular disassembly poly(lipoic acid) and antioxidative efficacies of F127@**1**-NP and F127@**2**-NP in t-BHP treated NRVMs. (**A**) Neonatal rat ventricular myocytes (NRVMs) were incubated with the indicated concentrations of fluorescently-labeled F127@**1**-NP and F127@**2**-NP for 3 h in DMEM plus 1% FCS and analyzed, after washings in NP-free medium, by flow cytofluorimetry. Data (Mean Fluorescence Intensity) are the mean of 3 independent experiments ± SE. (**B**) NRVMs were incubated with F127@**1**-NP and F127@**2**-NP (100 µg/mL) for the indicated time lapses and analyzed for their MFI by flow cytofluorimetry as above (data are mean ± SE, N = 3). * *p* < 0.001 vs. 5 min, # *p* < 0.05 vs. 5 min by two-way ANOVA with post hoc Tukey’s multiple comparison test. The red arrow indicates the pulse time with NPs (5 min) used for pulse–chase experiments described in panel (**C**). Cells were pulsed for 5 min as in panel (**B**), washed 3 times with PBS and further chased in cell medium with no NPs for the indicated times. Equivalent amounts of the buffer (Veh) or the fluorophore rhodamine (Rh) were used as controls. MFI acquired by spectrofluorimetry after cell recovery are expressed as relative values compared to the MFI one after the pulse time (taken as 1). Data are mean ± SE (N = 3). * *p* < 0.01 vs. 5 min, # *p* < 0.05 vs. 5 min, § *p* < 0.01 vs. NPs by two-way ANOVA with post hoc Tukey’s multiple comparison test. (**D**–**F**) NRVMs were preincubated with the shown concentrations of F127@**1**-NP and F127@**2**-NP for 1 h (**D**), 3 h (**E**) and 6 h (**F**), washed and further incubated with the CM-H_2_DCFDA. The ROS production was initiated with t-BHP (100 µM). ROS production rate (ROS production/time) is reported as a function of NP dose. Data are mean ± SE (N = 3).

Firstly, the uptake capacity of cardiomyocytes was evaluated. The fluorescence associated to neonatal rat ventricular myocytes (NRVMs) after incubation for 3 h with F127@**1**-NP was strikingly more intense than that due to F127@**2**-NP (Figure 4A). Time-dependent experiments revealed that, also with this cell line, the signal increase at early times showed a reduced capture rate, compared to macrophages at early times (<1 h), which was nevertheless similar for both F127@**1**-NP and F127@**2**-NP up to 60 min (Figure 4B). After this initial phase, fluorescence increased in both cases, but in a much more marked way in the presence of F127@**1**-NP compared to F127@**2**-NP. Pulse–chase experiments, again in line with what occurred in macrophages, demonstrated that the increase in fluorescence following internalization of F127@**1**-NP compared to F127@**2**-NP was due to cellular effects after internalization. However, in NRVMs the kinetics of fluorescence gain of internalized NPs, likely due to intracellular disassembly of the nanoparticles, was much more rapid (maximal effect after 0.5 h chase) (Figure 4C, Figures S11A,B and S12A) as compared to human macrophages maximal effect (after 3 h chase). NRVMs pretreated with NPs were protected from t-BHP induced ROS production, similarly to macrophages. In these cells, almost maximal protection was achieved following only a 1 h pre-incubation with the lower concentration (i.e., 25 µg/mL) (Figure 4D). However, in NRVMs the F127@**2**-NP was slightly less effective than F127@**1**-NP, likely due to a more marked differential degradation rate in macrophages. Nevertheless, the maximal protective effect was about 80%, and no further protection was observed by increasing the pre-incubation time point (Figure 4E,F).

### 3.7. Poly(Lipoic Acid) NPs Protect NRVMs from Ischemia/Reperfusion Oxidative Injury

**Figure 5:**
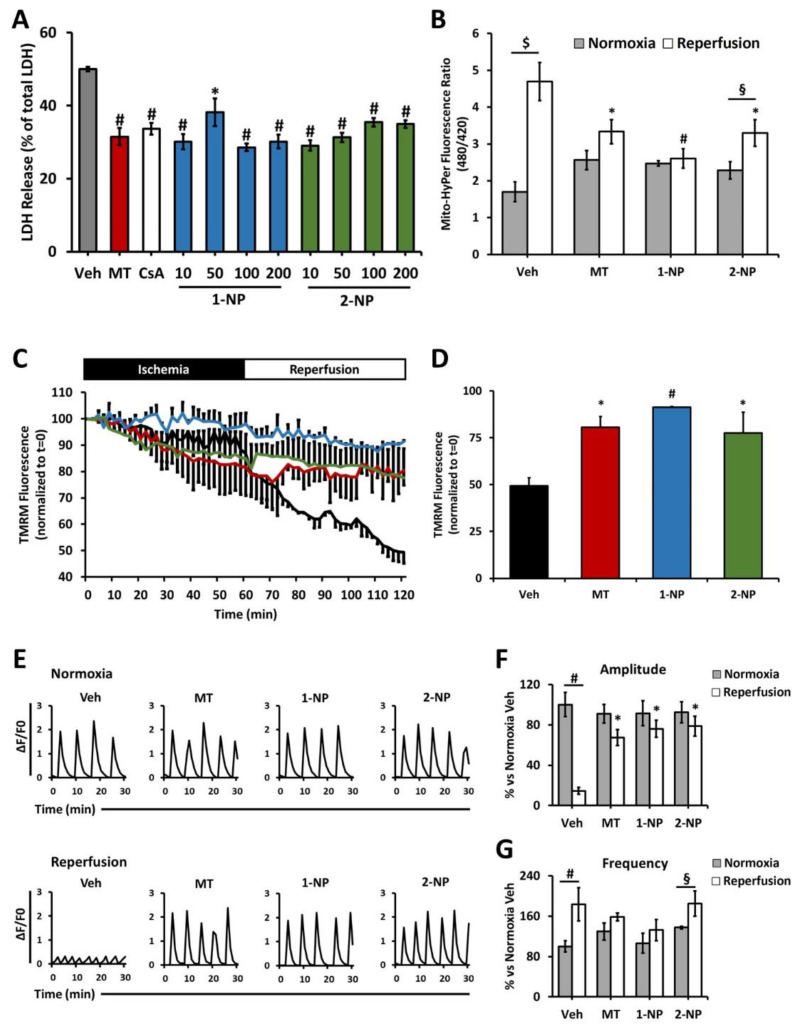
Poly(lipoic acid) NPs protect cardiomyocytes (RNVMs) from ischemia-reperfusion injury. (**A**) Cell death measured by LDH release in reoxygenation from isolated NRVMs. Cells were treated with different concentrations of either F127@**1**-NP or F127@**2**-NP. MitoTempo (MT, 10 µM) or cyclosporin A (CsA, 2 µM) were used as positive control. * *p* < 0.01 vs. Veh, # *p* < 0.001 vs. Veh by one-way ANOVA with post hoc Tukey’s multiple comparison test. (**B**) Mitochondrial H_2_O_2_ formation measured by Mito-HyPer in isolated NRVMs subjected to coverslip ischemia. Cells were treated with either F127@**1**-NP (100 µg/mL) or F127@**2**-NP (100 µg/mL). MitoTempo (MT 10 µM) was used as positive control. * *p* < 0.01 vs. Reoxygenation Veh, # *p* < 0.001 vs. Reoxygenation Veh, § *p* < 0.05, $ *p* < 0.001 by two-way ANOVA with post hoc Tukey’s multiple comparison test. (**C**) Mitochondrial membrane potential (ΔΨm) monitored by TMRM fluorescence in isolated NRVMs subjected to coverslip ischemia. Cells were treated with either F127@**1**-NP (100 µg/mL) or F127@**2**-NP (100 µg/mL). MitoTempo (MT 10 µM) was used as positive control. (**D**) ΔΨm was quantified after 60 min of simulated reperfusion. * *p* < 0.01 vs. Veh, # *p* < 0.001 vs. Veh by one-way ANOVA with post hoc Tukey’s multiple comparison test. (**E**) Representative intracellular [Ca^2+^] transients during normoxia (upper panel) and reperfusion (lower panel) in isolated NRVMs subjected to coverslip ischemia. Cells were treated with either F127@**1**-NP (100 µg/mL) or F127@**2**-NP (100 µg/mL). MitoTempo (MT 10 µM) was used as positive control. (**F**) Transient amplitude average in spontaneous beating NRVMs. * *p* < 0.001 vs. Veh Reperfusion, # *p* < 0.001 by two-way ANOVA with post hoc Tukey’s multiple comparison test. (**G**) Transient frequency average in spontaneous beating NRVMs. # *p* < 0.001, § *p* < 0.05 by two-way ANOVA with post hoc Tukey’s multiple comparison test. Approximately 30 cells were analyzed per condition in each experiment. For LDH release, approximately 3–4 wells were analyzed per condition in each experiment. All the experiments were performed at least three times using different animal preparations. Data are expressed as mean ± SEM.

Having observed poly(lipoic acid) NP capture by primary NRVMs and their subsequent protection from chemically-induced oxidative stress, we investigated the possible prevention of the reperfusion damage in an in vitro model of IRI. NRVMs were pretreated with increasing concentrations of F127@**1**-NP or F127@**2**-NP, ranging from 10 to 200 µg/mL, and cells were subjected to anoxia/reoxygenation (A/R) protocol to simulate IRI. Both F127@**1**-NP and F127@**2**-NP at concentrations as low as 10 µg/mL protected cardiomyocytes against reoxygenation-induced loss of viability as shown by a decrease in lactate dehydrogenase (LDH) release (% LDH release: ~50% untreated vs. ~30% treated) (Figure 5A). The degree of protection was comparable with that afforded by MitoTempo (MT) and cyclosporine A (CsA). Of note, both NPs did not induce loss of viability in either pre-treatment or anoxic phase (Figure S12C,D).

The burst of H_2_O_2_ production by mitochondria upon reperfusion was also inhibited by both F127@**1**-NP and F127@**2**-NP with an efficacy that was almost 100% in the case of the F127@**1**-NP and about 60% in the case of F127@**2**-NP, values comparable to that obtained with MT as a positive control (Figure 5B and Figure S12E). Consistently, the drop in the mitochondrial membrane potential subsequent to reperfusion was prevented by both F127@**1**-NP and F127@**2**-NP, with efficacies comparable to, if not better than, that displayed by MT (Figure 5C,D and Figure S12F). Eventually, the amplitude and the correct frequency of intracellular Ca^2+^ transients, which reflect the spontaneous contraction waves of NRVMs, were also preserved by both F127@**1**-NP and F127@**2**-NP preincubation, in spite of I/R damage (Figure 5E–G). In agreement with data obtained with t-BHP stress, the F127@**2**-NP were slightly less protective than the F127@**1**-NP against IRI.

## 4. Discussion

The present results highlight the antioxidative action of NPs made by cross-linked lipoic acid derivatives. Firstly, our study confirms the remarkable biocompatibility of these nanoparticles. Not only are they mainly composed of approved (F127) and endogenous (lipoic acid) building blocks, but they are also devoid of cellular toxicity and procoagulant activity, and complement activation. These results closely match those previously reported on these nanoparticles. In particular, it is remarkable to note the very small interaction with the plasma proteins, compared to other densely PEGylated nanoparticles. This resistance to protein adsorption may hence be attributed not only to the polymeric coating (PEG), but also to the influence that the specific core material used (polylipoic acid) may have on the coating molecules disposition and topology. This ability to escape plasma proteins is likely one of the factors at the basis of the high biocompatibility observed. 

The second remarkable property is the relevant uptake by cardiomyocytes. This is indeed not so far from that exerted by scavenging M2 macrophages (here used) for F127@**1**-NP (17–34%) and even equal for F127@**2**-NP (91–124%). Moreover, NPs cardiomyocytes uptake is sensibly greater than that of lymphocytes and epithelial cells (5% for F127@**1**-NP and 13% for F127@**2**-NP, compared to M2 macrophages). This finding corroborates and helps to explain the results of previous in vivo studies with rats, which demonstrated a fast (maximum at 1 h after injection) and selective accumulation of these nanoparticles in the heart. In particular, detailed microscopy investigations demonstrated that the nanoparticles were taken up by the cardiomyocytes present in heart tissues, as confirmed by the present findings. It is important to note that, different from PLGA nanoparticles, uptake occurs in healthy cells, and hence it is not dependent on the presence of an oxidative stress. This would in principle make it possible to also target the initial ROS burst at the onset of post-ischemic reperfusion.

After uptake, nanoparticles undergo degradation, as revealed by the pulse and chase experiments. This process can occur both in endosomes, by hydrolytic degradation of the ester groups, or in the cytoplasm, by concurrent hydrolysis of the ester groups and reduction of the disulfide bonds. The result of this degradation is the release of both the payload and lipoic acid. Interestingly, degradation occurs with different degrees of efficiency in the different cell lines. Indeed, degradation is clearly observed in macrophages and cardiomyocytes; it is also present, albeit less efficient, in monocytes and in HeLa cells, and it appears to be absent in PMGNs. Such differences likely reflect the differences in the respective cell microenvironments. In particular, degradation in cardiomyocytes is noticeably faster than in macrophages, with the consequent quick release of the protective species. The degradation rate is also strongly influenced by the structure of nanoparticles. Indeed, the more crosslinked nature of F127@**2**-NP makes them more resistant to degradation both upon exposition to thiols, and inside the cells. Other factors, in particular the extent of water penetration, can influence this process. Again, precursor **2** is more lipophilic than **1**, and should offer more resistance to water penetration. It is to be mentioned that our analysis, although revealing NP disassembly, cannot establish whether lipoic and hypothetical payloads are quantitatively released from NPs in cardiomyocytes and other cells.

Remarkably, the degradation rate is not clearly paralleled by ROS scavenging activity. In cardiomyocytes, and less clearly in Hela cells, faster degrading F127@**1**-NP shows a slightly better performance than F127@**2**-NP. On the other hand, in macrophages, the trend is reversed. This behavior suggests that degradation is not straightforwardly related to ROS scavenging perfomance, likely because the disulfide groups widespread in the nanoparticles’ cores are endowed with an antioxidant activity similar to that of lipoic acid.

Most remarkably, simulated reperfusion experiments on primary cardiomyocytes demonstrated a protective effect similar to that of leading cardioprotective compounds, highlighting the great potential of these nanoparticles.

## 5. Conclusions

At the end, not only do these soft NPs appear to be a good effective alternative to ceramic- or metal-based NPs as agents with intrinsic anti-oxidative effects, but they potentially allow for the development of more sophisticated drug systems. Indeed, while the NP matrix itself is active against ROS, it can at the same be loaded with pharmacologically active principles that are released only within cells, as Rhodamine was in this study. In addition, short-term accumulation of these NPs within the heart is independent of the physio pathological status of cells. This will allow them to act both as antioxidant particles and as a storage system of pharmacologically active principles, targeting simultaneously different stages of the IRI.

## Data Availability

Data is contained within the article and Supplementary Materials.

## Supplementary Materials

The following supporting information can be downloaded at: https://www.mdpi.com/article/10.3390/antiox11050907/s1, Table S1. Nanoparticle size determined by DLS of F127@**1**-NPs and F127@**2**-NPs; Figure S1. Characterization of nanoparticles; Figure S2. Lack of F127@**1**-NP and F127@**2**-NP cytotoxicity; Figure S3. Coagulation, complement activation and corona formation assays performed with F127@**1**-NP and F127@**2**-NP; Figure S4. Organic radical scavenging efficacy evaluation of F127@**1**-NP, individual components for NP synthesis and different known antioxidants; Figure S5. Antioxidant activity of NPs against t-BHP-derived ROS in human macrophages; Figure S6. F127@**1**-NP uptake by human purified monocytes and lymphocytes, monocyte-derived macrophages, PMNGs and RAW 264.7 and HeLa cells; Figure S7. Dose-response uptake of F127@**1**-NP by macrophages, monocytes and lymphocytes in the presence of different percentage of FCS, HS or HP; Figure S8. Capture of F127@**1**-NP and F127@**2**-NP by human macrophages in the presence of FCS or HS; Figure S9. F127@**1**-NP and F127@**2**-NP scavenging activity in human macrophages in response to increasing concentration of t-BHP; Figure S10. Capture and scavenging activity of F127@**1**-NP and F127@**2**-NP in HeLa cells; Figure S11. Comparison between chase phases of nanoparticles; Figure S12. Effects of nanoparticles on cardiomyocytes.
