# Supplementary material for: Nanoparticles Based on Cross-Linked Poly(Lipoic Acid) Protect Macrophages and Cardiomyocytes from Oxidative Stress and Ischemia Reperfusion Injury
Source: Antioxidants (Basel). 2022 May 5;11(5):907. doi: 10.3390/antiox11050907 (PMC9137738; doi:10.3390/antiox11050907)
Supplement: Supplementary file 1 [file antioxidants-11-00907-s001.zip › antioxidants-1692089-supplementary.pdf]

Nanoparticles based on cross-linked poly(lipoic acid) protect macrophages and cardiomyocytes from oxidative stress and ischemia reperfusion injury

Chiara Bellini [1,2,†,‡], Salvatore Antonucci [1,†], Lucía Morillas-Becerril [3,†,§], Sara Scarpa [1,2], Regina Tavano [1,2], Fabrizio Mancin [3], Fabio Di Lisa [1,*] and Emanuele Papini [1,2,*]

[1] Department of Biomedical Sciences, University of Padova, Via U. Bassi 58/b, 35121 Padova, Italy;
chiara.bellini@ttk.elte.hu (C.B.); salvatore.antonucci@unipd.it (S.A.);
sara.scarpa@studenti.unipd.it (S.S.); regina.tavano@unipd.it (R.T.)
[2] CRIBI—Centre for Innovative Biotechnology Research, University of Padova, Via U. Bassi 58/b, 35121 Padova, Italy
[3] Department of Chemical Sciences, University of Padova, Via F. Marzolo 1, 35121 Padova, Italy;
lucia.morillasbecerril@unipd.it (L.M.-B.); fabrizio.mancin@unipd.it (F.M.)
* Correspondence: fabio.dilisa@unipd.it (F.D.L.); papinie@bio.unipd.it (E.P.)
† These authors contributed equally.
‡ Present address: Hevesy György PhD School of Chemistry, Eötvös Loránd University, Pázmány Péter Sétány 1/A, 1117 Budapest, Hungary.
§ Present address: Department of Pharmaceutical Sciences, University of Milano, Via Golgi 19, 20133 Milano, Italy.

Table of contents

1. **General** 2
2. **Synthesis of disulphide monomers and dyes** 2
3. **General synthesis and characterization of poly-lipoic nanoparticles** 2
4. **In vitro characterization of poly-lipoic nanoparticles** 5

1. General

Chemical reagents were bought from Aldrich at highest commercial quality and used without further purification. Water was purified using a Milli-Q® and water purification system. Reactions were monitored by TLC developed on 0.25 mm Merck silica gel plates (60 F254) using UV light as visualizing agent and/or heating after staining with phosphomolybdic acid. Solvents were of analytical reagent grade, laboratory reagent grade or HPLC grade.

NMR spectra in the solution state were recorded on a AVIII 500 spectrometer (500 MHz for 1H frequency). UV-Vis absorption spectra were measured in methanol on a Varian Cary 50 spectrophotometer with 1 cm path length quartz cuvettes. Fluorescence spectra were measured in water or methanol on a Varian Cary Eclipse fluorescence spectrophotometer. Both the spectrophotometers were equipped with thermostatted cell holders. ESI-MS were recorded on Agilent Technologies 1100 Series system equipped with a binary pump (G1312A) and MSD SL Trap mass spectrometer (G2445D SL).

The hydrodynamic particle size (Dynamic Light Scattering, DLS) and Z-potential were measured with a Malvern Zetasizer Nano-S equipped with a HeNe laser (633nm) and a Peltier thermostatic system. Measurements were performed at 25 °C in water or PBS 10 mM buffer at pH 7. Transmission electron microscopy (TEM) was recorded on a FEI Tecnai G12 microscope operating at 100 kV. The images were registered with a OSIS Veleta 4K camera. Thermogravimetric analysis (TGA) was run on 50 µl nanoparticle samples using a Q5000 IR instrument from 25 to 1000 °C under a continuous air flow.

2. Synthesis of disulphide monomers and dyes

2.1. *Synthesis of octane-1,8-diyl bis(5-(1,2-dithiolan-3-yl) pentanoate)* (**1**). Monomer (**1**) was prepared as previously reported [1] yielding the product as yellow oil (84%).

2.2. *Synthesis of propane-1,2,3-tryl tris(5-(1,2-dithiolan-3-yl) pentanoate)* (**2**). Monomer (**2**) was prepared as previously reported [1] yielding the product as a yellow oil (73%).

2.3. *Synthesis of N-(9-(2-(4-(5-(1,2-dithiolan-3-yl) pentanoyl) piperazine-1-carbonyl) phenyl)-6-(diethylamino)-3H-xanthen-3-ylidene)-N-ethylethanaminium chloride* (**3**). Fluorophore (**3**) was prepared as previously reported [1] yielding the product as a pink solid (75%).

3. General synthesis and characterization of poly-lipoic nanoparticles

The lipoic acid-based nanoparticles were synthesized as previously reported by combination of nanoprecipitation and ring-opening disulfide exchange polymerization (Trzciński et al., 2021).

The acetone solution of the monomer (20 mL, 5 mg/mL) –or of mixed monomers- is added to an aqueous solution (1000 rpm) of PBS (pH 7.4, 100 mL, 2 mM of phosphate buffer) and Pluronic F-127 (100 mL, 20 mM) under stirring. Subsequently, a solution of 1-octanethiol (5 mg/ml) in acetone (10% v/v ratio with respect to the monomer solution) was added and the polymerization reaction was maintained for 90 min at room temperature. Then iodoacetamide (5 mg/mL) in acetone (10% v/v ratio respect to the monomer solution) was added to terminate the polymerization and the reaction was stirred for another 30 minutes. The formed nanoparticles were collected by centrifugation and washed with H_2O. Final product was stored at 4^oC as water suspension.

Table S1. Nanoparticle size determined by DLS of F127@**1**-NPs and F127@**2**-NPs.

	Size (nm)	***PDI***
F127@**1**-NPs	169.6	0.040
	181.0	0.052
	184.4	0.041
	174.9	0.012
	187.9	0.063
F127@**2**-NPs	150.4	0.089
	161.0	0.059
	146.1	0.073
	143.0	0.054
	141.8	0.048
	139.3	0.131

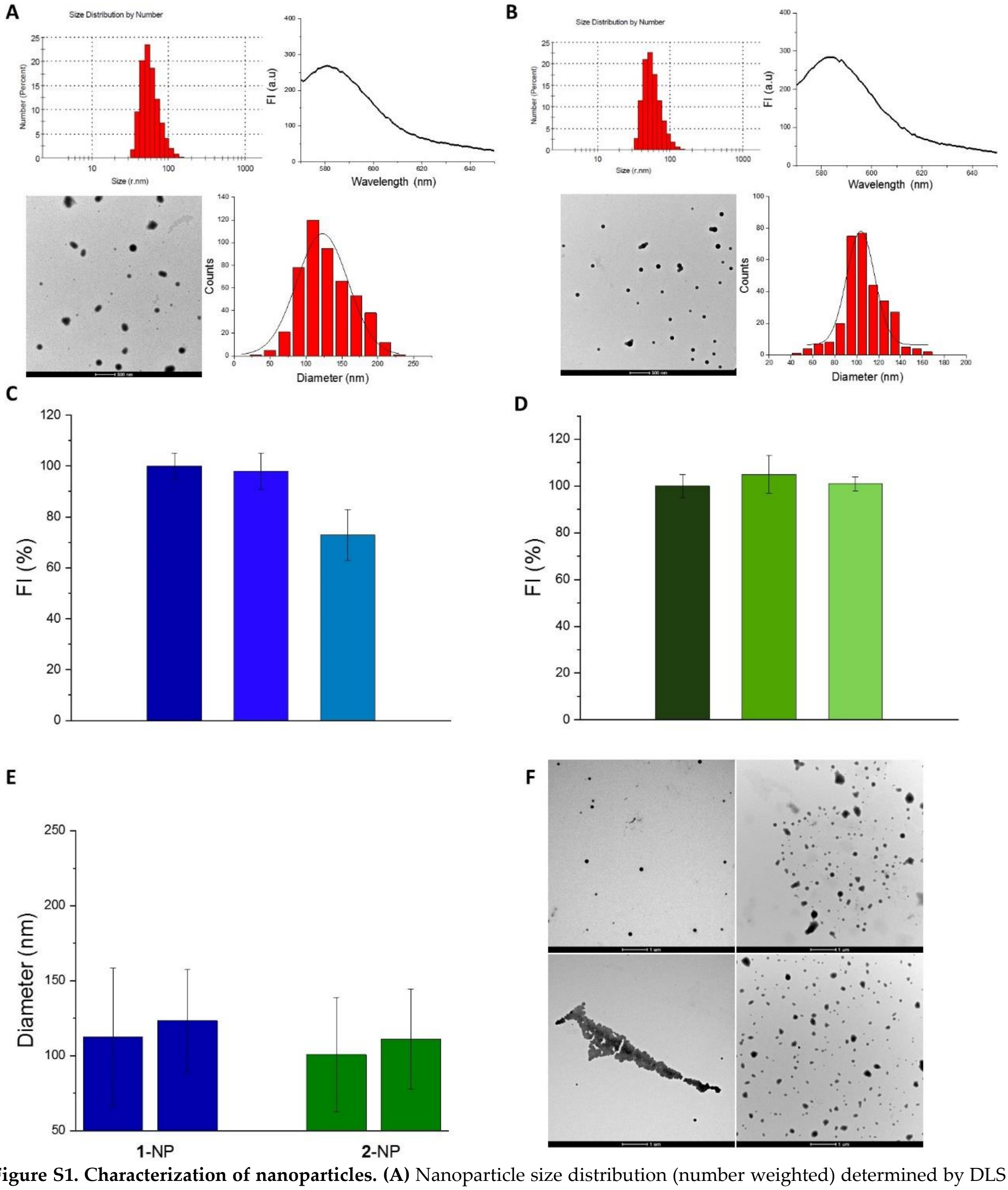

Figure S1. Characterization of nanoparticles. (A) Nanoparticle size distribution (number weighted) determined by DLS of F127@**1**-NPs doped with **3** (average size 113 nm, PDI: 0.165 measured by DLS), fluorescence intensity (arbitrary units) of F127@**1**-NPs doped with **3**. Concentration of rhodamine was calculated by extrapolation of a calibration curve, TEM image, size distribution of F127@**1**-NPs doped with **3** and fitting curve parameters (average diameter = 122 nm, σ = 72 nm). **(B)** Nanoparticle size distribution (number weighted) determined by DLS of F127@**2**-NPs doped with **3** (average size 101 nm, PDI: 0.143 measured by DLS), fluorescence intensity (arbitrary units) of F127@**2**-NPs doped with **3**. Concentration of rhodamine was calculated by extrapolation of a calibration curve: TEM image, size distribution of F127@**2**-NPs doped with **3** and fitting curve parameters (average diameter = 104 nm, σ = 27 nm). **(C)** Time dependent fluorescence intensity percentage of fluorescent nanoparticles of F127@**1**-NPs doped with **3** at day 0 (dark blue), after 2 weeks (blue) and after 3 weeks (light blue). **(D)** Time dependent fluorescence intensity percentage of fluorescent nanoparticles of F127@**2**-NPs doped with **3** at day 0 (dark green), after 2 weeks (green) and after 3 weeks (light green). **(E)** Hydrodynamic size (F127@**1**-

NPs blue bars, F127@**2**-NPs green bars) over the period of 22 days. **(F)** TEM micrographs of F127@**1**-NPs after the synthesis (top left) and after 22 days (top right). B) F127@**2**-NPs after synthesis (bottom left) and after 22 days (bottom right).

4. In vitro characterization of poly-lipoic nanoparticles

4.1. *MTS assay.* To determine NPs cytotoxicity, cells (1x104 cells/well HeLa, 1x105 cells/well human macrophages) were seeded in a 96-well plate (Falcon). The day of the experiment cells were treated with different concentrations of NPs (up to 400 μg/ml) in cellular medium supplemented with 10% FCS (v/v); after 24h, the medium was removed, and cells were incubated with 110 μl of 0.1% v/v MTS in medium solution (Promega) at 37°C until color development. Absorbance was detected at 492 nm and the percentage of alive cells was calculated with respect to non-treated cells.

4.2. *Hemolysis assay.* Human erythrocytes were obtained from human blood of healthy volunteers after elimination of buffy coats, washed three times and resuspended in PBS at a final concentration of 10% (v/v). Then, they were incubated with NPs at different concentrations (up to 400 μg/ml) for 2 hours at 37°C. After, samples were centrifuged (1500 rpm, 5 min) and supernatant absorbance determined at 540 nm; data were expressed as percentage with respect to positive control (human erythrocytes incubated with water).

4.3. *Agglutination assay.* Human erythrocytes were isolated as described above (see Hemolysis section). 50 μl of 1% (v/v) erythrocytes suspension (with or without 50% HS supplement) was dispensed onto a 96-well U-shaped plates and incubated with 50 μl of NPs solutions at different concentration (up to 400 μg/ml) for 2 hours at room temperature. After, results for agglutination were visually assessed.

4.4. *Plasma clotting time.* 77 μl of human plasma were added to 100 μl of NPs or Ludox® at different concentrations (up to 400 μg/ml) in 150 mM NaCl solution in a 96 well microtiter plate (Sarstedt) and coagulation was started by the addition of 23 μl of 150 mM CaCl2 solution. Changes in optical density at 405 nm were detected every 60s for 60 times at 37°C. Therefore, the time required to reach half maximal absorbance increase (t½) was calculated.

4.5. *C3a detection*. To control complement activity, 25 μl of human sera was treated with 6.25 μl of zymosan (25 mg/ml, Sigma, prepared as described by manufacturer's instructions) for 30 minutes at 37°C; reaction was stopped with 25 mM EDTA. To assess complement activation induced by NPs, 25 μl of human sera were incubated with 6.25 μl of NPs at different concentration (up to 400 μg/ml) for 30 minutes at 37°C. Then, 1.6 μl of each sample were mixed with 38.4 μl of water and 6.7 μl of loading sample buffer without β-mercaptoethanol; and 15 μl of sample were loaded onto a 12% gel. Proteins were then transferred to PVDF membrane which, after saturation with TBS-T containing 1% nonfat milk at room temperature for 1 h, were further treated in the same solution with antibody against C3a (Calbiochem, 1:1000) overnight at 4°C. Membrane was washed three times with TBS/0.1% (v/v) Tween 20 and treated with secondary HRP-(Calbiochem)conjugated Abs. Proteins were detected by Huvitec (Eppendorf) and band densitometry was performed using ImageJ software.

4.6. *NP protein corona determination by SDS-PAGE combined with silver staining protocol.* NPs (50 μg/ml) were incubated at 37°C for 30 min in RPMI-1640 medium supplemented with 10%, 20% or 50% HS (or HP or FCS). NPs were washed three times with 1 mL of ice-cold PBS and then recovered by centrifugation (30 min, 12000 rpm at 4°C). NP pellets were dissolved in 25 μl of loading sample buffer 1X (300 mM Tris-HCl, 12% SDS, 0.4% bromophenol blue, 40% glycerol, 5% βMe). After being heated at 95°C for 5 min, equal volumes (12 μl) of samples were subjected to SDS-PAGE in 12% acrylamide gel. For silver staining, gels were fixed for 30 min in 50% v/v, methanol 10% (v/v) acetic acid; and then incubated for 15 min in 5% v/v methanol, 1% v/v acetic acid, washed three times with water, and exposed for 90 s to thiosulfate solution (200 μg/ml Na2S2O3 pentahydrate). After extensive washing with water, gels were incubated in the dark for 30 min with 0.2 g/l AgNO3, rinsed, and developed for 5–15 min with a solution containing 60 mg/ml Na2CO3, 4 μg/ml Na2S2O3 pentahydrate, and 0.01875% v/v formaldehyde. Reaction was stopped with 6% (v/v) acetic acid.

4.7. *Cells.* Human lymphocytes, monocytes and PMNGs were purified by buffy coats of healthy donors, by means of by two sequential centrifugations on Ficoll and Percoll (HE Healthcare) gradients, as previously described (Fedeli et al., 2013)); Raw 264.7 cells were maintained in Dulbecco's Modified Eagle Medium (DMEM, Gibco) supplemented with 10% FCS (v/v) (Euroclone®) and antibiotics (penicillin and streptomycin, Invitrogen) at 37°C in a humidified atmosphere containing 5% (v/v) CO_2; cells were split every 2–3 days. Cells were incubated with different concentrations of nanoparticles as described in the main text (both at 37°C or at 0°C) and they were analyzed by FACS.

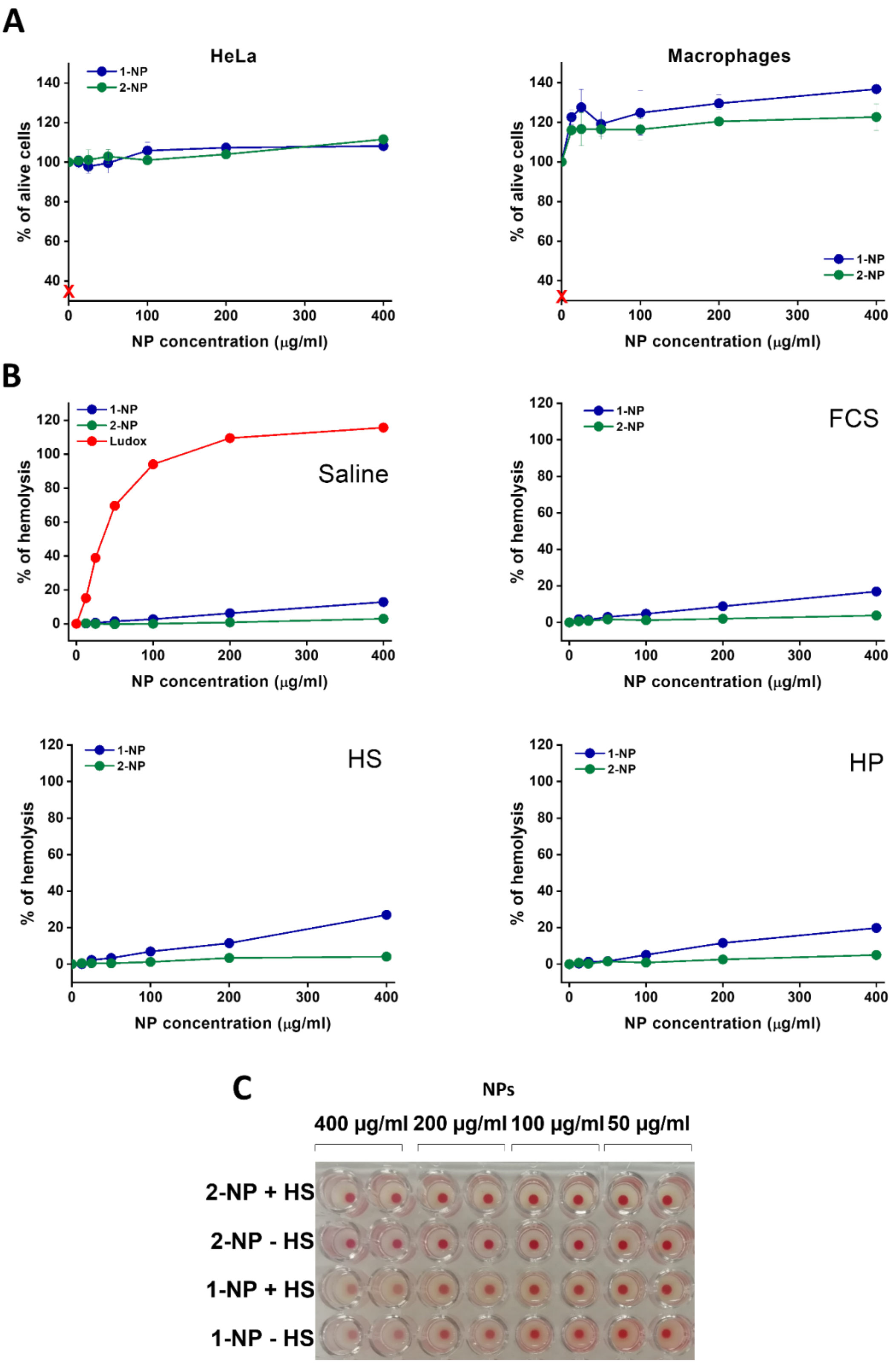

Figure S2. Lack of F127@1-NP and F127@2-NP cytotoxicity. (A) HeLa (left panel) or human macrophages (right panel) were incubated for 24 hours with F127@**1**-NP and F127@**2**-NP at the indicated concentrations in RPMI 10% FCS before MTS assay. The red cross indicates the effect of distilled water, as positive control. Data are the mean ± SE (N=3). **(B)** Purified human red blood cells were treated for 2 hours with F127@**1**-NP and F127@**2**-NP, or with Ludox® SiO_2-NPs as positive control, at the indicated concentrations in PBS, 25% FCS, HS or HP, as indicated. Data are the mean of a representative experiment run in duplicate (mean ± SE). **(C)** Human red blood cells were incubated in PBS, plus or minus HS in the presence of the indicated concentrations of F127@**1**-NP and F127@**2**-NP for 2h in U shaped wells plates. The lack of agglutination is indicated by the well-defined dot in the bottom of wells.

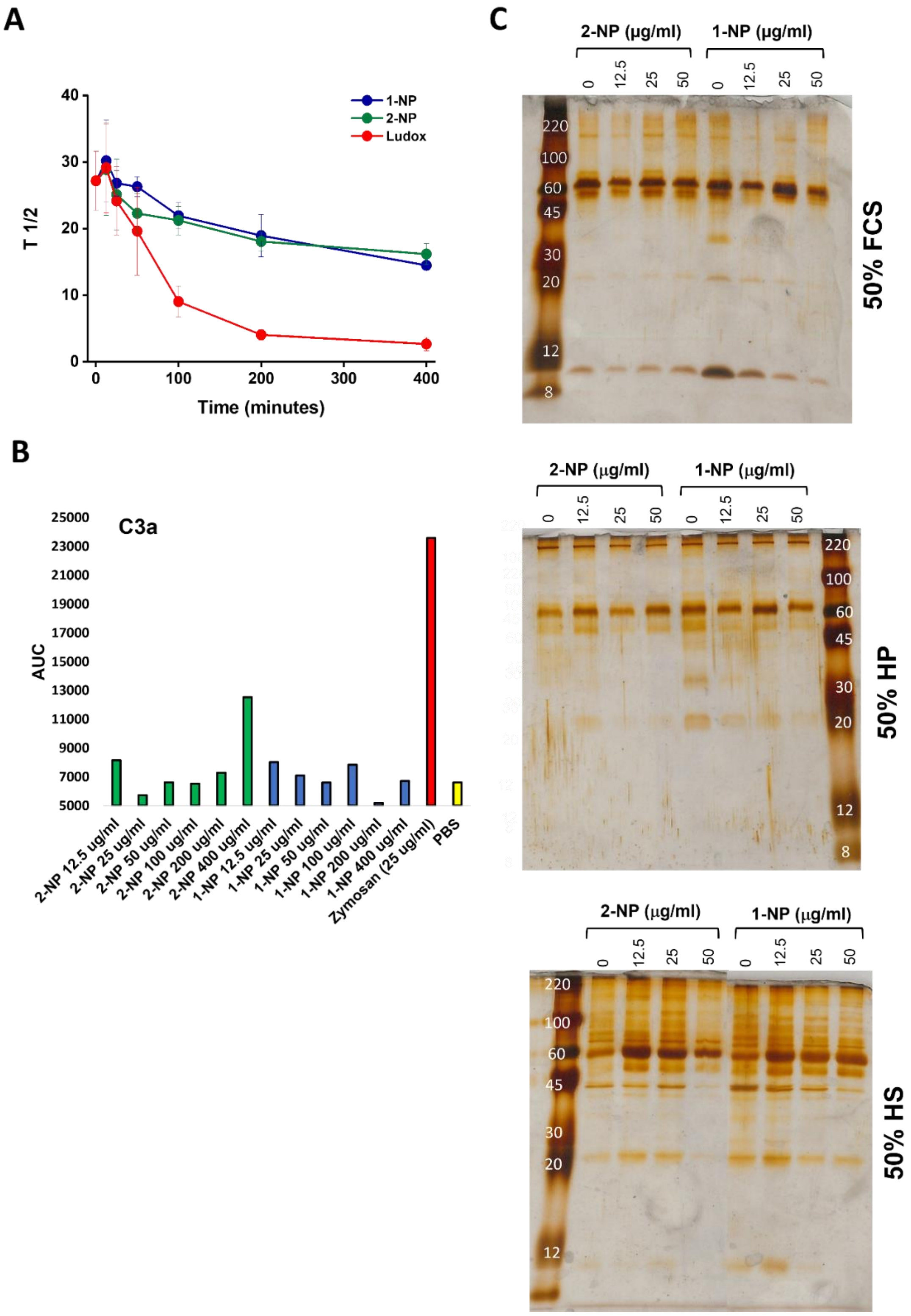

Figure S3. Coagulation, complement activation and corona formation assays performed with F127@1-NP and F127@2-NP. (A) F127@**1**-NP and F127@**2**-NP and Ludox® SiO2-NPs as positive control, were added to citrated HP at the indicated concentrations. At time zero, concentrated Ca2+ was added to the solution to final 17 mM and the following kinetics of light scattering increase was monitored. The time giving half-maximal coagulation is reported as a function of NP doses. Data are the mean ±S E (N=3). **(B)** F127@**1**-NP and F127@**2**-NP or Zymosan as positive control were incubated at 37°C for 30 min with HS as the indicated concentrations. C3a formation was measured by WB using C3a specific antibodies. Data correspond to C3a band densitometry of a representative experiment out of three. **(C)** F127@**1**-NP and F127@**2**-NP at the indicated concentrations were incubated at 37°C in FCS, HS and HP (50% v/v) for 20 min, washed three times by centrifugation with ice cold PBS, pH7,4 and eventually resuspended in LSB and subjected to SDS-PAGE. Bands were silver stained.

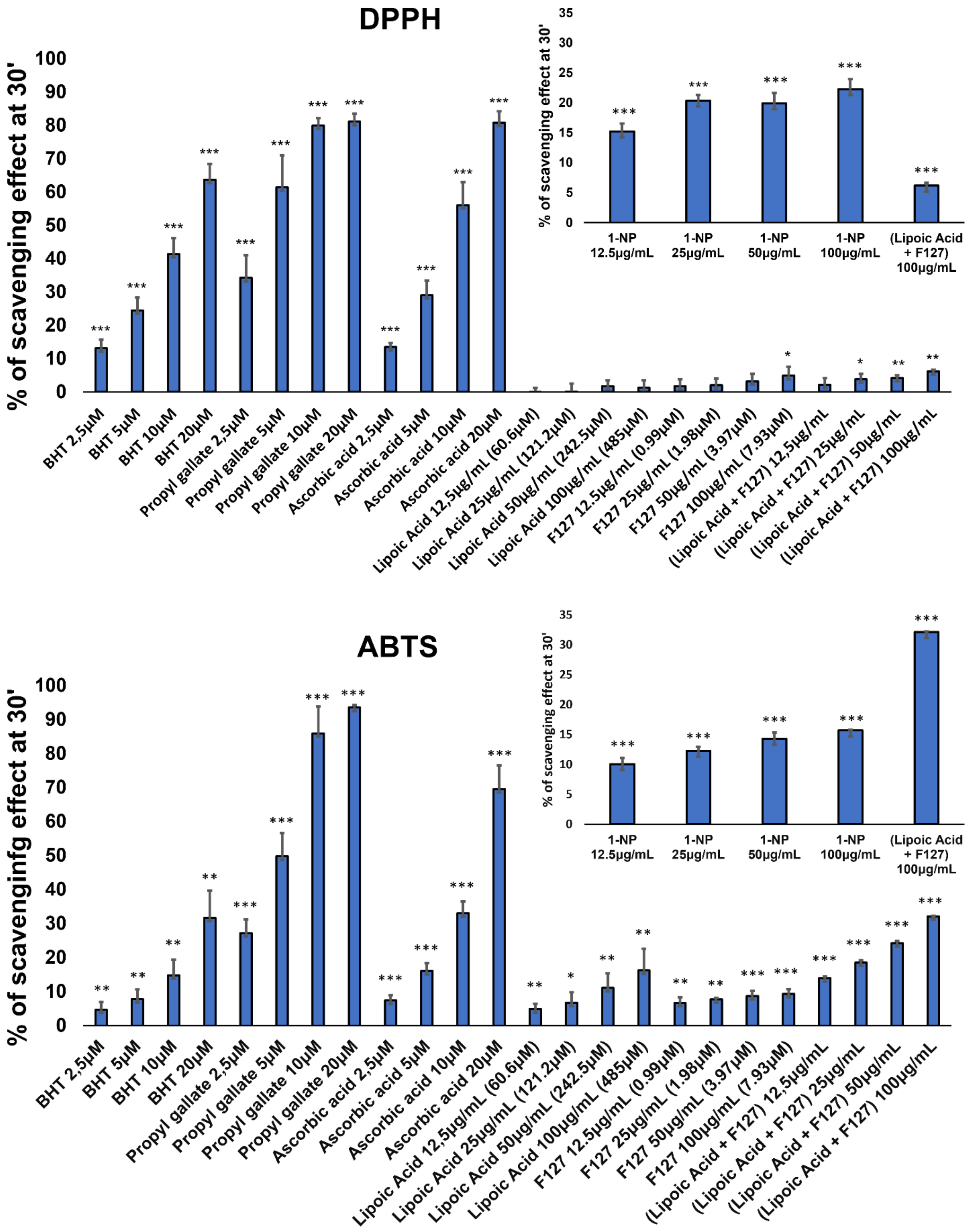

Figure S4. Organic radical scavenging efficacy evaluation of F127@1-NP, individual components for NP synthesis and different known antioxidants. The indicated concentrations of BHT, Propyl gallate, Ascorbic acid, Lipoic acid, F127 and F127@**1**-NP were incubated with DPPH (A) or ABTS (B) and the % of neutralization was estimated spectroscopically after 30 min incubation at 37°C. Data are means ±SE (N=4).

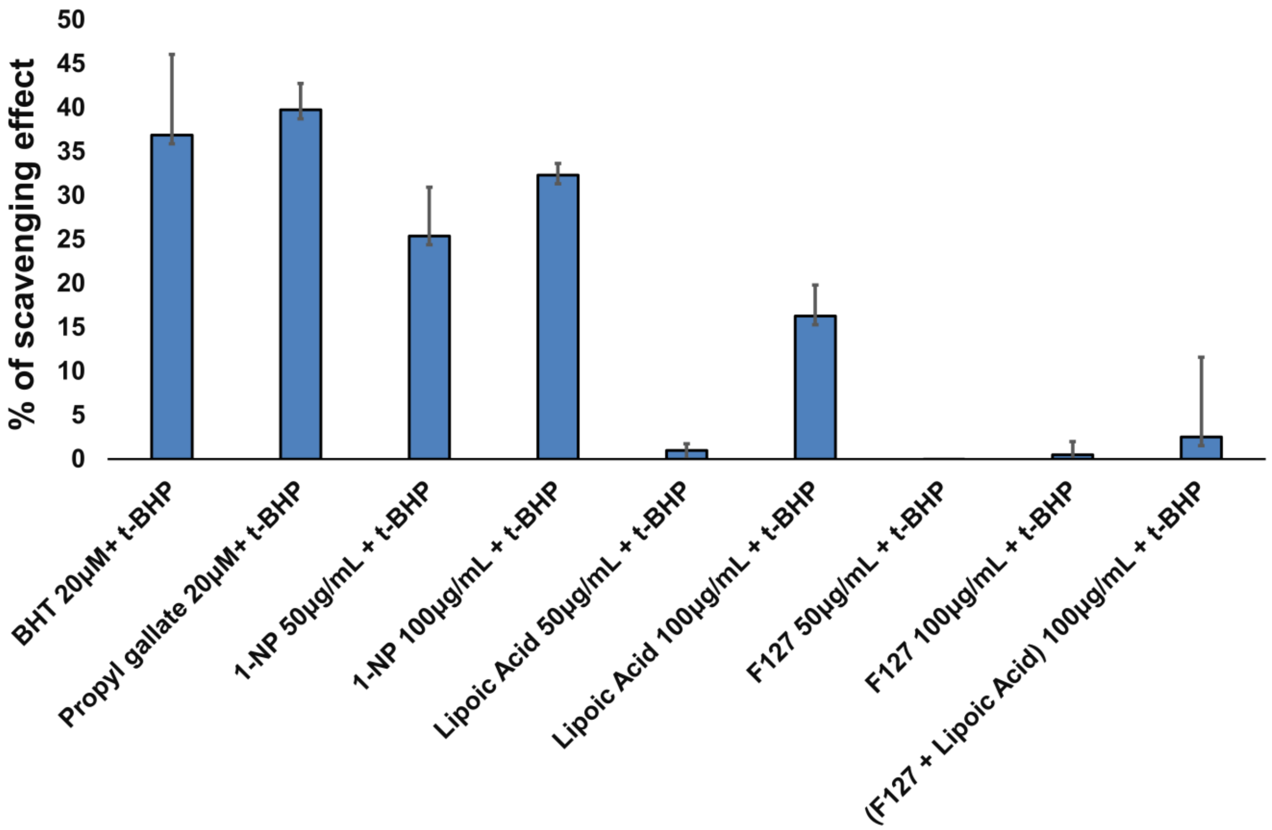

Figure S5. Antioxidant activity of NPs against t-BHP-derived ROS in human macrophages. Macrophages were incubated with the indicated antioxidant concentrations and t-BHP (0.5mM) for 1 h at 37°C. Cells were then washed and incubated with 25 µM Carbossi-H_2DCFDA for 20′ at 37°C. After 2 washes in PBS, cells were resuspended in FACS buffer and the fluorescence was measured by flow cytometry.

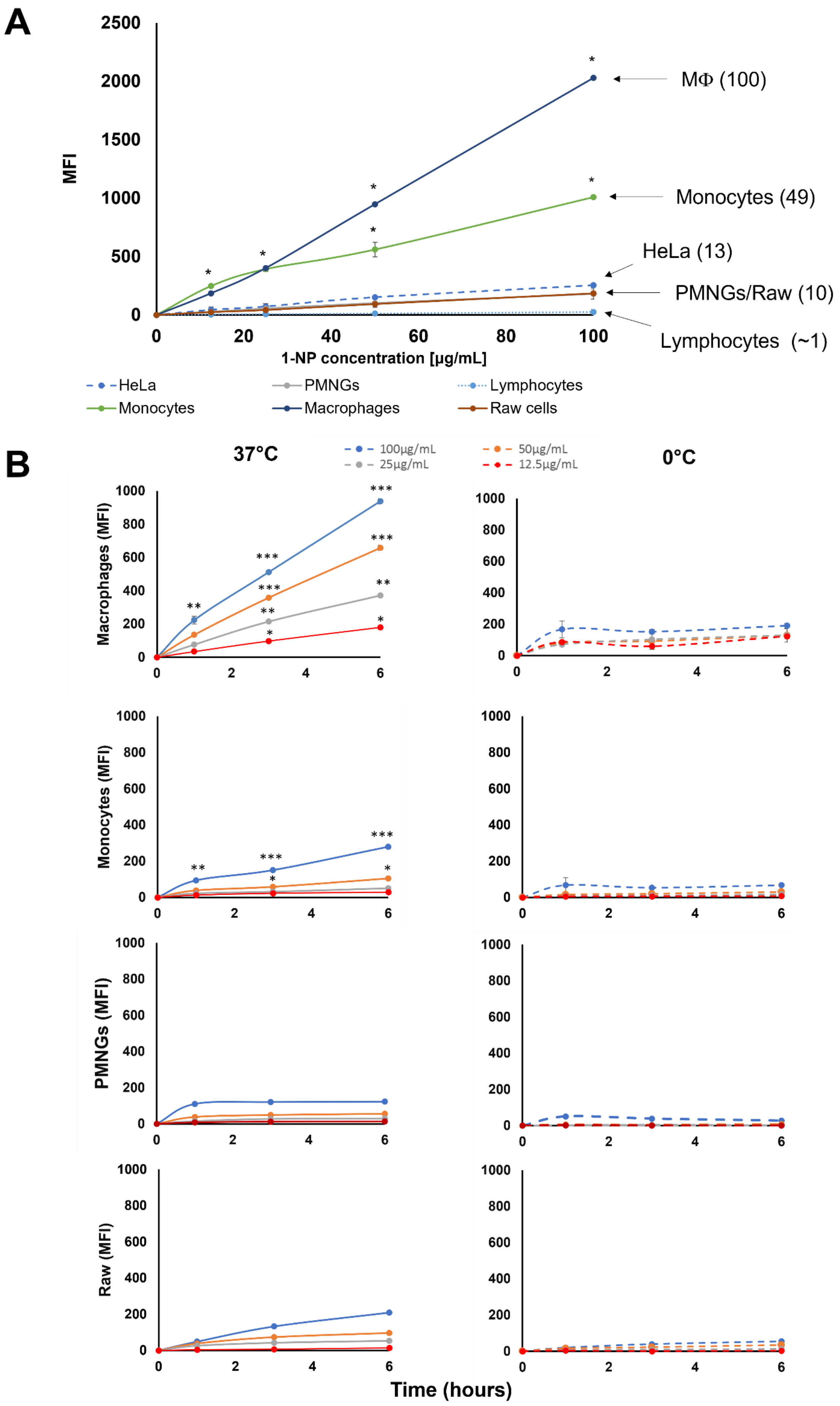
A
B
MFI
1-NP concentration [µg/mL]
MΦ (100)
Monocytes (49)
HeLa (13)
PMNGs/Raw (10)
Lymphocytes (~1)
HeLa
PMNGs
Lymphocytes
Monocytes
Macrophages
Raw cells
37°C
0°C
100µg/mL
50µg/mL
25µg/mL
12.5µg/mL
Macrophages (MFI)
Monocytes (MFI)
PMNGs (MFI)
Raw (MFI)
Time (hours)

Figure S6. F127@1-NP uptake by human purified monocytes and lymphocytes, monocyte-derived macrophages, PMNGs and RAW 264.7 and HeLa cells. (A) Cells were treated with the indicated concentrations of fluorescent F127@**1**-NP for 24h at 37°C; then they were washed in PBS, resuspended in FACS buffer and fluorescence associated to cells was measured by flow cytometry. Data are means ±S E (N=4). *p<0.05 with respect not treated cells; **(B)** Macrophages, monocytes, PMNGs or Raw 264.7 cells were incubated with the indicated rhodamine-labelled F127@**1**-NP concentrations for up to 6 hours at 37°C or at 0°C; cells were then washed with PBS, resuspended in FACS buffer and analyzed by low cytometry. Data are means ±S E (N=4). *p<0.05, **p<0.01 and ***p<0.001 with respect not treated cells.

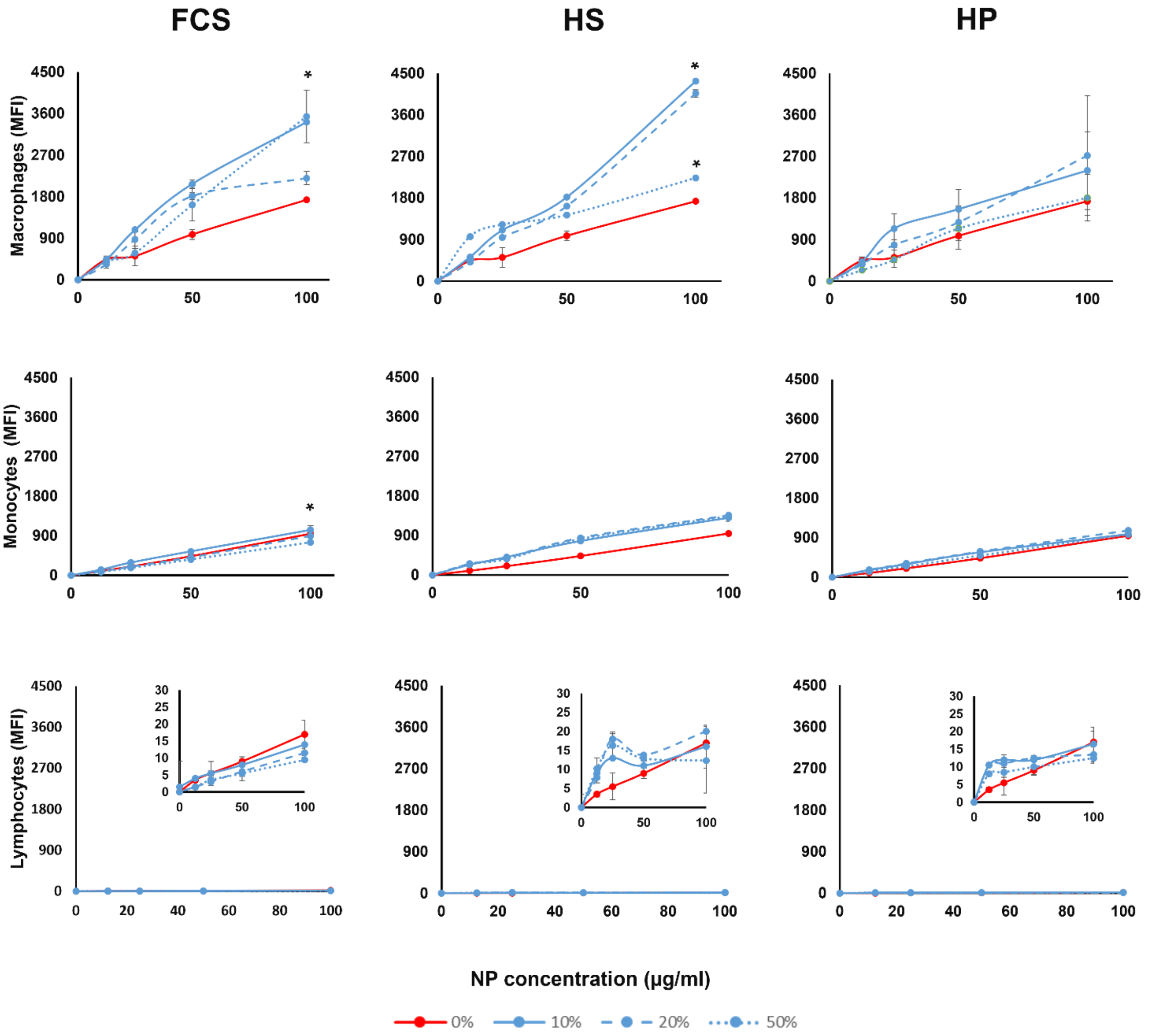

Figure S7. Dose-response uptake of F127@1-NP by macrophages, monocytes and lymphocytes in the presence of different percentage of FCS, HS or HP. Cells were incubated for 24h with the indicated NP concentrations and percentage of FCS, HS or HP. Cells were then washed with PBS, resuspended in FACS buffer and analyzed by low cytometry. Data are means ±S E (N=3). *p<0.05 with respect cells treated with the same NP concentration but in absence of serum or plasma.

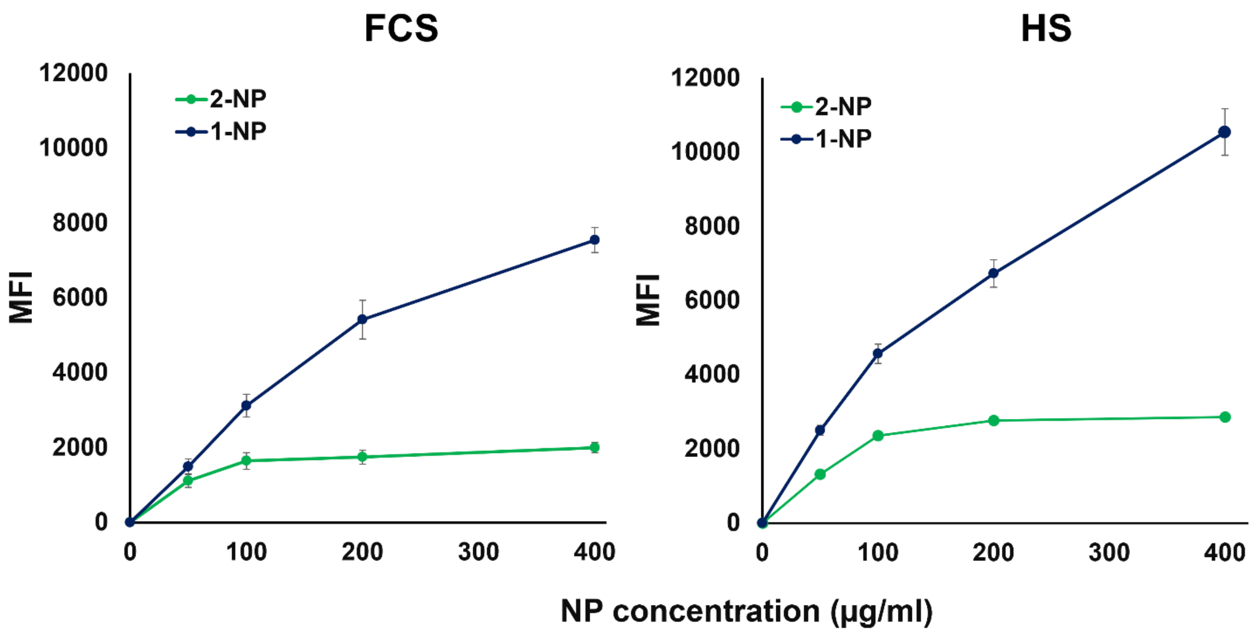

Figure S8. Capture of F127@1-NP and F127@2-NP by human macrophages in the presence of FCS or HS. Human macrophages were incubated with the indicated doses of fluorescently labelled F127@**1**-NP and F127@**2**-NP, for 3 hours in DMEM plus 10% FCS or 10% HS and analyzed, after washings in NP-free medium, by flow cytofluorimetry. Data (Mean Fluorescence intensity) are the mean of 4 independent experiments ± SE

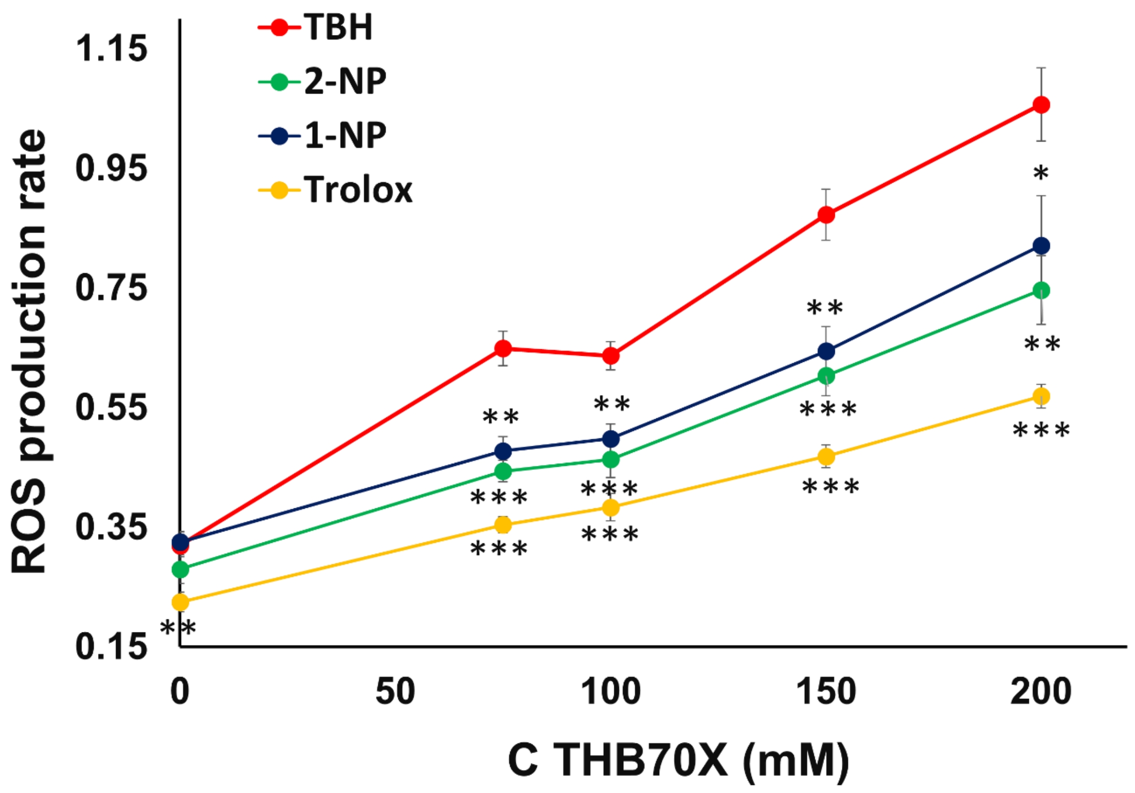

Figure S9. F127@1-NP and F127@2-NP scavenging activity in human macrophages in response to increasing concentration of t-BHP. Human macrophages were treated with F127@**1**-NP and F127@**2**-NP (200 ug/ml) for 3 hours and then incubated with the ROS-sensitive probe. Afterward, cells were treated with the shown t-BHP concentrations to investigate the scavenging activity of the NPs in response to different oxidative stress conditions. ROS production rate

(ROS production/time) is reported as a function of t-BHP concentration. Data are mean ± SE (N=4). Statistical significance of the difference compared with no NP samples: * t< 0.05; ** t<001; *** t< 0.001

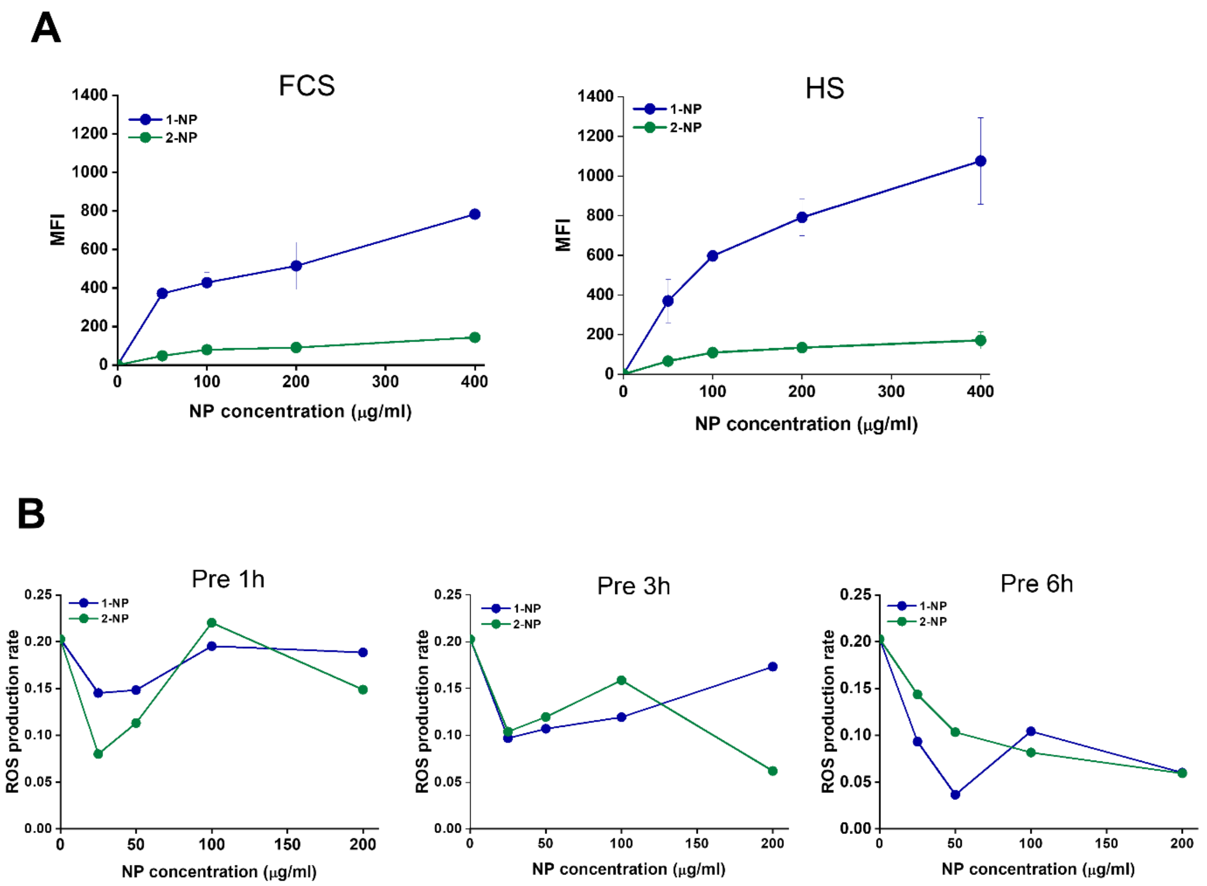

Figure S10. **Capture and scavenging activity of F127@1-NP and F127@2-NP in HeLa cells. (A)** HeLa cells were incubated for 3 hours at 37°C with the indicated concentrations of F127@**1**-NP and F127@**2**-NP; cells were analyzed by flow cytometry, after washing with PBS. Data (Mean Fluorescence intensity) are the mean of 3 independent experiments ±SE. **(B)** HeLa cells were treated with the shown concentrations of F127@**1**-NP and F127@**2**-NP for 1, 3 and 6 hours. Cells were incubated with the ROS-sensitive probe and then ROS production was induced with t-BHP (200 µM). ROS production rate (ROS production/time) is reported as a function of NP dose. Data are mean ± SE (N=4). Statistical significance of the difference compared with no NP samples: * t< 0.05; ** t<001; *** t< 0.001

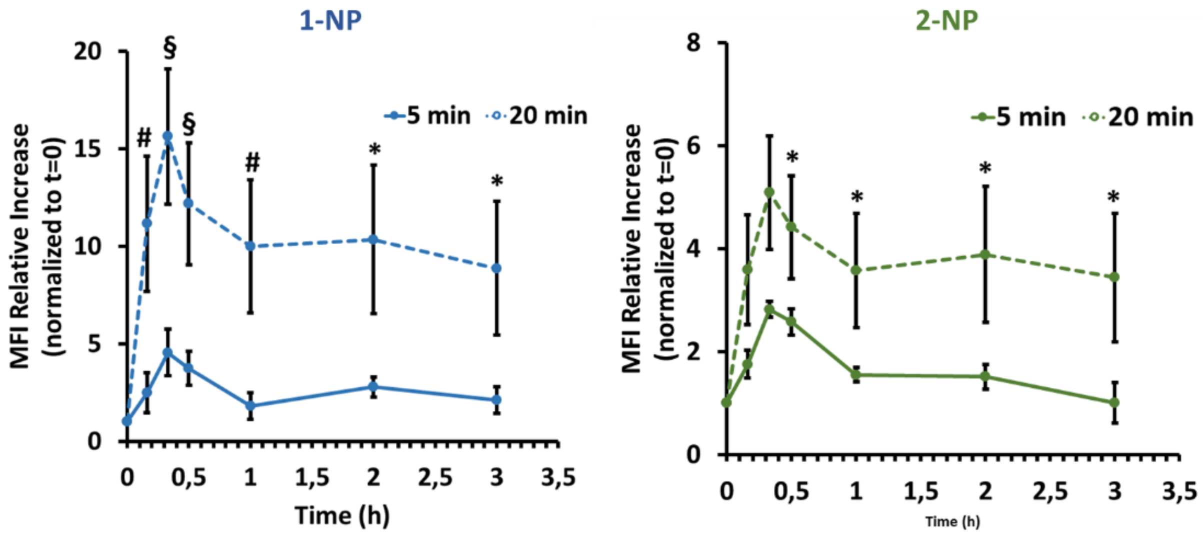

(A) (B)

Figure S11. (**A**) Comparison between chase phases of F127@**1**-NP evaluated after either 5 min or 20 min of pulse phase. $^*p < 0.05$, $^\#p < 0.01$, $^\S p < 0.001$ by two-way ANOVA with *post hoc* Tukey's multiple comparison test. (**B**) Comparison between chase phases of F127@**2**-NP evaluated after either 5 min or 20 min of pulse phase. $^*p < 0.05$ by two-way ANOVA with *post hoc* Tukey's multiple comparison test. Approximately 6 wells were analyzed per condition in each experiment. All the experiments were performed at least two times using different animal preparations. Data are expressed as mean ± SEM.

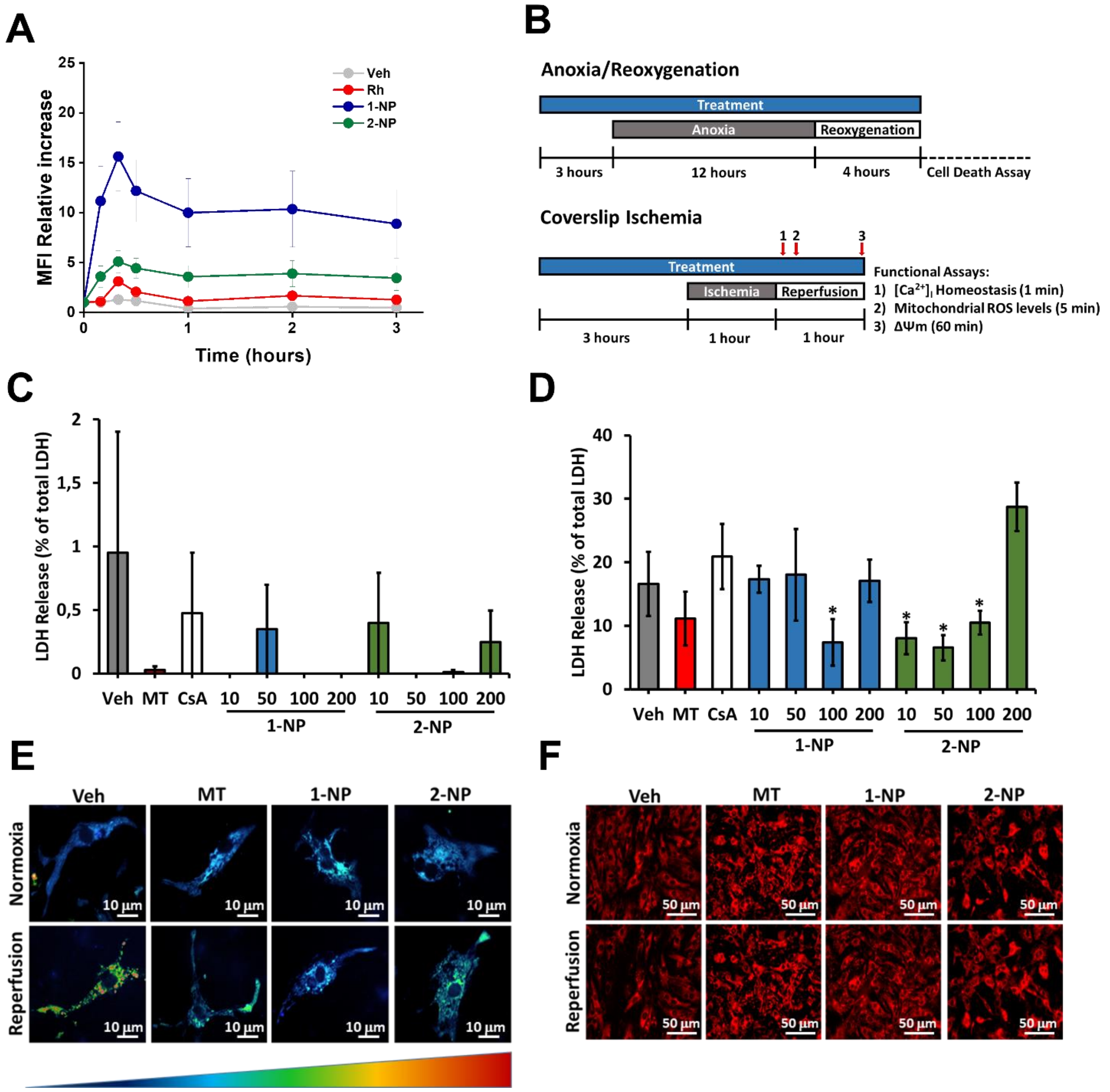

Figure S12. Effects of nanoparticles on cardiomyocytes. (**A**) *Chase:* NRVMs have been exposed to 5 min of incubation with 100 µg/ml of either F127@**1**-NP or F127@**2**-NP conjugated with rhodamine and the fluorescence has been monitored at different time points. Unconjugated rhodamine has been used as negative control. $^*p < 0.01$ vs 5 min D, $^\#p < 0.05$, $^\S p < 0.01$ by two-way ANOVA with *post hoc* Tukey's multiple comparison test. (**B**) Graphical representation of the two protocols adopted to simulate ischemia/reperfusion *in vitro*. (**C**,**D**) Cell death measured by LDH released during (**C**) the pre-treatment phase or (**D**) the anoxic phase from isolated NRVMs. Cells have been treated with different concentrations of either F127@**1**-NP or F127@**2**-NP nanoparticles. MitoTempo (MT, 10 µM) or cyclosporin A (CsA, 2 µM) have been used as positive control. $^*p < 0.05$ vs T200 by one-way ANOVA with *post hoc* Tukey's multiple comparison test. (**E**) Representative figures of mitochondrial H_2O_2 formation measured by Mito-HyPer in isolated NRVMs subjected to coverslip ischemia. Cells have been treated with either F127@**1**-NP (100 µg/ml) or F127@**2**-NP (100 µg/ml). MitoTempo (MT 10 µM) has been used as positive control. (**F**) Representative figures of (ΔΨm) monitored by TMRM fluorescence in isolated NRVMs subjected to coverslip ischemia. Cells have been treated with either F127@**1**-NP (100 µg/ml) or F127@**2**-NP (100 µg/ml). MitoTempo (MT 10 µM) has been used as positive control. Approximately 30 cells were analyzed per condition in each experiment. For chase assay approximately 6 wells were analyzed per condition in each experiment. For LDH release approximately 3-4 wells were analyzed per condition in each experiment. All the experiments were performed at least three times using different animal preparations. Data are expressed as mean ± SEM.
